# A Practical Guide to Sliding and Surface Semilandmarks in Morphometric Analyses

**DOI:** 10.1093/iob/obz016

**Published:** 2019-07-05

**Authors:** C Bardua, R N Felice, A Watanabe, A -C Fabre, A Goswami

**Affiliations:** 1 Department of Life Sciences, Natural History Museum, Cromwell Rd, Kensington, London, SW7 5BD, UK; 2 Department of Genetics, Evolution & Environment, University College London, Gower St, Bloomsbury, London, WC1E 6BT, UK; 3 Centre for Integrative Anatomy, Department of Cell and Developmental Biology, University College London, Gower St, Bloomsbury, London, WC1E 6BT, UK; 4 Department of Anatomy, New York Institute of Technology College of Osteopathic Medicine, Northern Blvd, Old Westbury, NY 11568, USA; 5 Division of Paleontology, American Museum of Natural History, Central Park West at 79th Street, New York, NY 10024, USA

## Abstract

Advances in imaging technologies, such as computed tomography (CT) and surface scanning, have facilitated the rapid generation of large datasets of high-resolution three-dimensional (3D) specimen reconstructions in recent years. The wealth of phenotypic information available from these datasets has the potential to inform our understanding of morphological variation and evolution. However, the ever-increasing ease of compiling 3D datasets has created an urgent need for sophisticated methods of capturing high-density shape data that reflect the biological complexity in form. Landmarks often do not take full advantage of the rich shape information available from high-resolution 3D specimen reconstructions, as they are typically restricted to sutures or processes that can be reliably identified across specimens and exclude most of the surface morphology. The development of sliding and surface semilandmark techniques has greatly enhanced the quantification of shape, but their application to diverse datasets can be challenging, especially when dealing with the variable absence of some regions within a structure. Using comprehensive 3D datasets of crania that span the entire clades of birds, squamates and caecilians, we demonstrate methods for capturing morphology across incredibly diverse shapes. We detail many of the difficulties associated with applying semilandmarks to comparable regions across highly disparate structures, and provide solutions to some of these challenges, while considering the consequences of decisions one makes in applying these approaches. Finally, we analyze the benefits of high-density sliding semilandmark approaches over landmark-only studies for capturing shape across diverse organisms and discuss the promise of these approaches for the study of organismal form.

## Synopsis

Fortschritte in der Bildgebungstechnologie wie Computertomographie (CT) und Oberflächenerfassung haben in den letzten Jahren die schnelle Generierung großer Datensätze von hochauflösenden 3D-Probenrekonstruktionen ermöglicht. Die Fülle an phänotypischen Informationen, die aus diesen Datensätzen verfügbar ist, kann unser Verständnis der morphologischen Variation und Evolution beeinflussen. Die immer einfachere Erstellung von 3D-Datensätzen hat jedoch zu einem dringenden Bedarf an ausgeklügelten Methoden zur Erfassung von Gestaltdaten in hoher Dichte geführt, die die biologische Komplexität in der Form widerspiegeln. Landmarken nutzen häufig die umfangreichen Forminformationen, die bei hochauflösenden 3D-Probenkonstruktionen zur Verfügung stehen, nicht in vollem Umfang aus, da sie sich in der Regel auf Nähte oder Fortsätze beschränken, die zuverlässig über mehrere Proben hinweg identifiziert werden können und einen Großteil der Oberflächenmorphologie ausschließen. Die Entwicklung von Gleit- und Oberflächen-Semilandmarken-Techniken (sliding and surface semilandmarks) hat die Quantifizierung der Form erheblich verbessert, ihre Anwendung auf vielfältige Datensätze kann jedoch eine Herausforderung darstellen, insbesondere beim Umgang mit variabler Abwesenheit einiger Bereiche innerhalb einer Struktur. Anhand von umfassenden 3D-Datensätzen von Schädeln, die sich über die vollständigen Kladen der Vögel, Squamata und Caecilia erstrecken, zeigen wir Methoden zur Erfassung der Morphologie über unglaublich diverse Formen hinweg. Wir gehen auf viele der Schwierigkeiten ein, die mit der Anwendung von Semilandmarken auf vergleichbare Regionen über sehr ungleiche Strukturen hinweg zusammenhängen, und bieten Lösungen für einige dieser Herausforderungen unter Berücksichtigung der Konsequenzen von Entscheidungen, die bei der Anwendung dieser Ansätze getroffen werden. Abschließend analysieren wir die Vorteile von gleitenden Semilandmarken in hoher Dichte gegenüber reinen Landmarkenstudien zur Erfassung der Gestalt über diverse Organismen hinweg und diskutieren die Aussichten dieser Ansätze für die Untersuchung der organismischen Form. Translated to German by F Klimm (frederike.klimm@biologie.uni-freiburg.de)

Um guia prático para demarcação de semi pontos de referência de superfície e de deslizamento em análises morfométricas Os avanços nas tecnologias de imagem, como a tomografia computadorizada (CT) e a varredura de superfície, facilitaram a rápida geração de grandes conjuntos de dados de reconstruções de espécimes 3D de alta resolução nos últimos anos. A riqueza de informações fenotípicas disponíveis nesses conjuntos de dados tem o potencial de informar nossa compreensão da variação e evolução morfológica. No entanto, a facilidade cada vez maior de compilar conjuntos de dados 3D criou uma necessidade urgente de métodos sofisticados para a captura de dados de alta densidade que reflitam a complexidade biológica na forma. Os pontos de referência morfológicos geralmente não capturam o máximo das informações sobre a morfologia disponíveis nas reconstruções de espécimes 3D em alta resolução, pois normalmente são restritas a suturas ou processos que podem ser identificados de forma confiável em diferentes espécimes, excluindo a maior parte da morfologia de superfície. O desenvolvimento de técnicas de deslizamento e de semi pontos de referência de superfíce melhorou muito a quantificação da forma, mas sua aplicação a diversos conjuntos de dados pode ser um desafio, especialmente quando algumas regiões dentro de uma estrutura são ausentes. Usando conjuntos de dados tridimensionais abrangentes do crânio, abrangendo todos os clados de pássaros, lagartos Squamata e cecílias, nós demonstramos métodos para captura da morfologia em formas incrivelmente diversas. Nós detalhamos muitas das dificuldades associadas à aplicação de semi pontos de referência em regiões comparáveis de estruturas altamente díspares, e fornecemos soluções para alguns desses desafios, enquanto consideramos as consequências das decisões tomadas na aplicação dessas abordagens. Finalmente, analisamos os benefícios das abordagens de deslizamento do semi pontos de referência em alta densidade para capturar a forma em diversos organismos e discutir a promessa dessas abordagens para o estudo da forma do organismo. Translated to Portuguese by Diego Vaz (dbistonvaz@vims.edu)

## Introduction

 Recent advances in specimen digitization have led to rapid accumulation of high-resolution phenotypic data. Specifically, computed tomography (CT) and surface scanning have allowed the efficient creation of digital specimen reconstructions, providing rich morphological datasets with relative ease (Davies et al. 2017). This revolution in high quality data has driven demand for new methods which more comprehensively capture phenotypic diversity (disparity), ultimately permitting more accurate and precise representation of organismal morphology (Goswami et al. 2019).

Quantifying morphology has been a cornerstone of biology for centuries, from Cope’s analyses of body size evolution across living and fossil taxa (Cope 1887) to D’Arcy Thompson’s splines of shape deformation through ontogeny (Thompson 1917). Through this long history, there has been great attention paid to improving the accuracy of representations of organismal form and incorporating those representations into models of evolutionary and developmental dynamics. Over the last few decades, the field of morphometry has blossomed through the development and extensions of the geometric morphometric paradigm (Bookstein 1991; Rohlf and Marcus 1993; Dryden and Mardia 1998; Lele and Richtsmeier 2001; Adams et al. 2004; Zelditch et al. 2004; Gunz et al. 2005; Slice 2005; Mitteroecker and Gunz 2009). Geometric morphometric methods (Bookstein 1991; Zelditch et al. 2004; Lawing and Polly 2010; Adams et al. 2013) typically involve the use of two- or three-dimensional (2D or 3D) coordinate points to quantify shape that is independent of differences in position, rotation, and isometry. Numerous recent reviews cover the breadth and utility of geometric morphometric methods, which are now widely used across the biological sciences, from translational studies of developmental anomalies (e.g., Waddington et al. 2017) to detailed estimates of long-extinct ancestral morphologies (Da Silva et al. 2018; Felice and Goswami 2018; Watanabe et al. 2019). The expansion of the geometric morphometric toolkit and increasing ease of applying these approaches to diverse datasets has greatly enhanced the study of organismal morphology.

However, landmark-based geometric morphometrics still suffers from limitations in its representation of organismal form, specifically due to reliance on merely discrete points for comparisons across specimens. These discrete landmarks bring two major constraints. First, they are typically limited in number due to their reliance on clear biological homology across specimens (levels of homology and landmark categorization are discussed further below). These points of clear homology can quickly diminish in numbers even in closely related taxa, meaning that representations of morphology become increasingly poor when studying more subtle variations in form (e.g., intraspecific variation) or when other major sources of morphological differences are not characterized by existing landmarks. This is especially a problem when many biological structures lack the discrete points of clear homology that define most geometric morphometric landmarks. Studies of limb bones, for example, will often leave large regions unsampled by any landmarks. This loss of morphological information is clearly undesirable as geometric morphometrics continues to expand in applications to deep-time and broad comparative studies. The second drawback is that landmarks, by definition, fail to characterize the shape between landmarks. Even structures formed from many elements and that provide many sutures and processes for consistent placement of landmarks will bear regions without any discrete points, such as the cranial vault. To address these issues, recent years have seen further expansions of geometric morphometrics to include the use of semilandmarks to capture shape along curves and surfaces (Gunz et al. 2005; Gunz and Mitteroecker 2013), pseudolandmark methods (Boyer et al. 2011, 2015), or landmark-free methods (Pomidor et al. 2016). These approaches greatly improve the representation of morphology and alleviate both of the issues noted above, by densely sampling the regions that may not have many discrete points of homology within or between them but represent homologous structures across specimens.

Pseudolandmark methods have been developed to transform surface meshes into clouds of points that are then subjected to a blind Procrustes superimposition (e.g., cPDist, Boyer et al. 2011, auto3dgm Boyer et al. 2015). These methods remove subjectivity in placing landmarks, as well as massively reducing time required to gather morphometric data. However, pseudolandmark methods do not allow the allocation of points into different biologically defined regions and cannot ensure points are positioned in anatomically equivalent positions throughout a dataset, limiting the ability to link patterns of variance to specific mechanisms of interest (e.g., developmental tissues). For a discussion surrounding the limitations of pseudolandmark methods, see Gao et al. (2017), and for similar methods see a landmark-free approach (Pomidor et al. 2016) and eigensurface analysis (which transforms each specimen’s mesh into a grid of regularly spaced points, Polly and MacLeod 2008). The ability to retain correspondence between data points is important for many morphological studies, especially to compare morphology across different regions of a structure, as in studies of modularity, and thus sliding semilandmark approaches may be particularly useful for studies that are concerned with questions other than differences in overall shape among specimens.

Semilandmarks (Bookstein 1991; Gunz et al. 2005; Gunz and Mitteroecker 2013) offer, in a sense, an intermediate characterization between homology-based landmark approaches and homology-free pseudolandmark methods. They maintain comparability of biologically informed parts across specimens by optimizing fit, by minimizing either bending energy or Procrustes distance and resulting in geometric homology of semilandmarks (Bookstein 1991; Gunz et al. 2005, 2009). Curve sliding semilandmarks define outlines, such as the margins of bones or fins and anatomical ridges, so they represent a significant increase in shape capture compared to landmark-only datasets (Bookstein 1997). These semilandmarks have been used successfully to quantify a vast array of organismal morphology, including beak shape (Cooney et al. 2017), the inner ear of xenarthrans (Billet et al. 2015), fish fins (Larouche et al. 2018), turtle shells (Vitek 2018), ostracod valves (Wrozyna et al. 2016), ant bodies (Yazdi 2014), and human corpus callosum shape (Bookstein et al. 2002). The further addition of surface sliding semilandmarks (defining entire surfaces which are demarcated by landmarks and curves) results in an even denser, more comprehensive quantification of shape. In particular, combining landmarks, curve sliding semilandmarks, and surface semilandmarks allows for defining regions within a structure as well as capturing the complex morphology of 3D surfaces (Adams et al. 2013).

The application of 3D surface semilandmarks (in addition to landmarks and curve semilandmarks) is only a recent advancement in the field of geometric morphometrics (Gunz et al. 2005; Mitteroecker and Gunz 2009; Gunz and Mitteroecker 2013), but already its utility has been demonstrated through the detailed quantification of shape across a wide array of taxa. However, while curve sliding semilandmarks are placed manually onto specimens, the application of surface sliding semilandmarks using a template is less intuitive. With this approach, surface sliding semilandmarks are not placed manually onto each specimen; they are applied to surfaces in a semi-automated approach, constrained in their placement by landmarks and curves delimiting the boundaries of each region onto which they are applied (although see Niewoehner 2005 for an alternative, manual, method). This method has been successfully applied to capture the morphology of, for example, bivalve scallops (Sherratt et al. 2016), hominin crania (Gunz et al. 2009), head shape of snakes (Segall et al. 2016), the skull (Dumont et al. 2015) and forelimb (Fabre et al. 2013a, 2013b, 2014, 2015) of musteloid carnivorans, the skull and mandible of the greater white-toothed shrew (Cornette et al. 2013, 2015) and primates (Fabre et al. 2018b), the femur of sciuromorph rodents (Wölfer et al. 2019), the long bones of mustelids (Botton-Divet et al. 2016) and primates (Fabre et al. 2017, 2018a, 2019), the brain of New World monkeys (Aristide et al. 2016), and the palate of human children (Pavonia et al. 2017). Methods combining curve and surface sliding semilandmarks are, therefore, starting to be applied to a wide range of datasets and are emerging as one of the most promising approaches for taking advantage of the high-resolution information on morphology offered by 3D image data.

Despite being used in analyses for over a decade, detailed descriptions of sliding semilandmark methods, in particular as applied to surfaces, tend to focus on the underlying mathematics rather than on the step-by-step procedure for implementing these approaches. Consequently, this lack of guidance has prevented the collection of surface semilandmark data from becoming a more widespread and implemented method. For this reason, here we provide a practical guide to 3D sliding and surface semilandmark data collection, in combination with 3D landmarks, using recently developed toolkits. We describe in detail the steps and decisions required for applying this high-dimensional data approach, drawing on examples from intergeneric datasets that span limbed vertebrate diversity. We identify several challenges we encountered from applying this procedure to datasets spanning considerable disparity in form, provide a range of solutions, and assess the consequences of different approaches for troubleshooting. As these high-density approaches will be useful for many researchers taking advantage of the new possibilities allowed by 3D datasets, we hope that this guide will prove useful and informative for the next generation of studies quantifying organismal form in 3D.

## Brief overview of landmarking approach

**Fig. 1:**
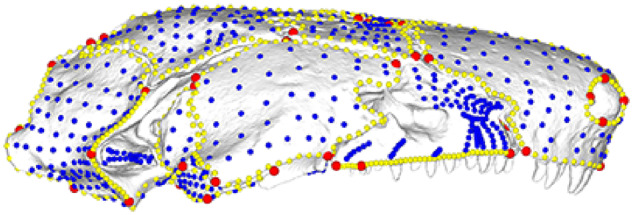
Annotated 3D version of this figure available at: https://sketchfab.com/3d-models/add35e2e8af94839b1f577bfcee32e54. Landmark and semilandmark data displayed on the caecilian *Siphonops annulatus* BMNH 1956.1.15.88. Points are colored as follows: landmarks (red), sliding semilandmarks (“curve points,” yellow), and surface semilandmarks (“surface points,” blue). For information regarding each cranial region, see Bardua et al. (2019). BMNH, Natural History Museum, London, UK.

**Fig. 2:**
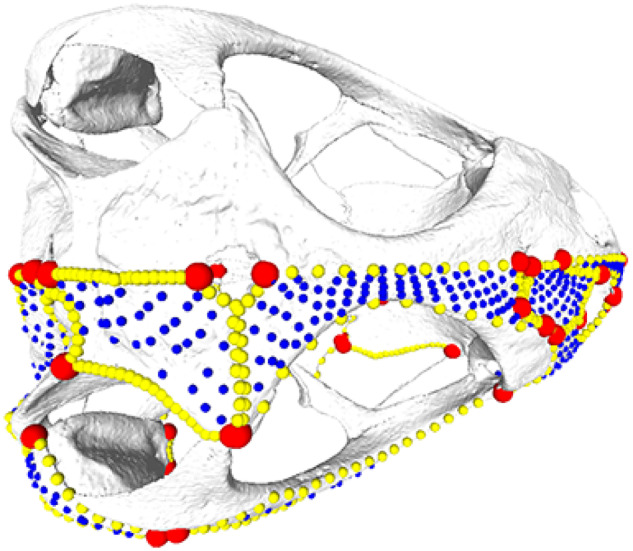
Annotated 3D version of this figure available at: https://sketchfab.com/3d-models/f6c4e6a649be48079a8747b80a52e40d. Landmark and semilandmark data displayed on the squamate *Sceloporus variabilis* FMNH 122866. Points are colored as follows: landmarks (red), sliding semilandmarks (“curves points,” yellow), and surface semilandmarks (“surface points,” blue). For information regarding each cranial region, see Watanabe et al. (2019). FMNH, Field Museum of Natural History, Chicago, IL, USA.

**Fig. 3:**
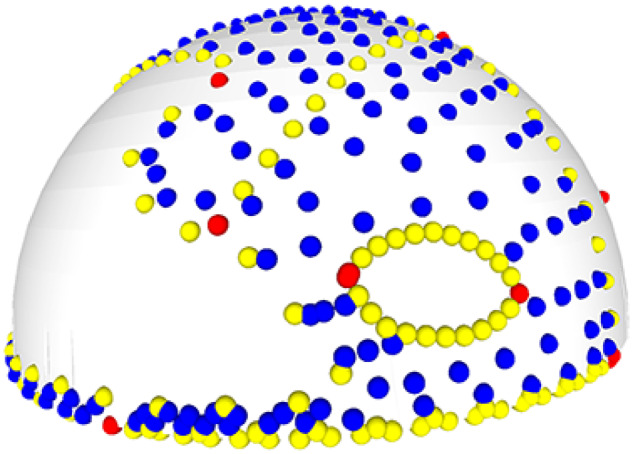
Annotated 3D version of this figure available at: https://sketchfab.com/3d-models/88cf8af1d00343729ffb7d4627a08df7. An example of a template used to apply surface semilandmarks onto specimens. Here, landmarks (red), sliding semilandmarks (yellow), and surface semilandmarks (blue) are manually placed onto a hemispherical mesh. This template is used to apply the surface semilandmarks onto specimens. This template was used in a recent study of bird crania (Felice and Goswami 2018).

The method discussed in this paper involves the manual placement of anatomically-defined landmarks and sliding semilandmarks (the latter forming “curves” between landmarks (Gunz et al. 2005)) onto specimens, defining regions of interest on a structure (Figs. 1 and 2). Surface semilandmarks are semi-automatically projected onto each specimen using a template (Gunz et al. 2005; Schlager 2017). The construction of the template requires a surface mesh (the “template mesh”) onto which landmarks and curves are placed which match those of the specimens, with the addition of surface semilandmarks that will be projected semi-automatically onto each specimen during the patching step (Fig. 3). Landmarks and sliding semilandmarks are placed onto specimens and the template using IDAV Landmark Editor v.3.6 (Wiley et al. 2005) or Checkpoint (Stratovan, Davis, CA, USA), using the “single point” and “curve” options, respectively. These landmarks and curves delimit different regions within the structure. Surface semilandmarks are then manually placed onto each region of the template (using the “single point” option in Landmark Editor or Checkpoint), and the template is used in a semi-automated procedure in R (R Core Team 2017) for placing these surface points onto each region of each specimen. Surface points can be generated automatically for entire surfaces (e.g., Aristide et al. 2016), but this approach is not as transferable for structures with multiple regions because the distribution and number of points in each cranial region cannot be controlled. During the patching procedure, the template is warped to the shape of each specimen and the surface points are projected onto each specimen. The points are expanded outwards by a specified amount along their normals to prevent these points from being stuck inside the mesh surfaces. Then, they are “deflated” along their normals until they come in contact with a mesh surface. The surface points are then slid to minimize total bending energy of a thin plate spline (TPS) across all specimens. Subdividing a structure allows the researcher to investigate a wide-range of shape-related questions, such as exploring how specific regions of morphology have evolved. This patching procedure is implemented in the R packages *Morpho* (Schlager 2016) and *geomorph* (Adams and Otárola-Castillo 2013), as well as in Edgewarp (Bookstein and Green 1994), Mathematica routines (Wolfram Research, Champaign, Illinois), MorphoDig (http://morphomuseum.com/morphodig (Lebrun and Orliac 2017)), and EVAN toolbox (Phillips et al. 2010), although only *Morpho* and *geomorph* will be discussed here. For a practical comparison of *Morpho* and Edgewarp, see Botton-Divet et al. (2015). Please refer to the *Morpho* package literature (Schlager 2017) for detailed code to implement the patching and sliding procedures. The main functions discussed here are for the patching procedure (placePatch) and a sliding procedure (slider3d) in the *Morpho* R package (Schlager 2017). Table 1 lists the main programs and packages mentioned in this guide, and Table 2 lists the terms used and their definitions.

**Table 1:** Useful software and functions

Name	Specific function	Use
IDAV Landmark (or Stratovan Checkpoint) (Wiley et al. 2005)	Single points	Placing landmarks on specimens, placing landmarks and surface semilandmarks on template
	Curves	Placing sliding semilandmarks on specimens and template
Meshlab (Cignoni et al. 2008)	Quadric Edge Collapse Decimation	Mesh decimation
	Create New Mesh Layer	Simple template creation
Geomagic Wrap (3D Systems, Rock Hill)	Fill single	Filling in surface holes and sutures (after material has first been manually removed to create a break in the mesh surface)
	Remove spikes	Remove rugosity, smooth surface of mesh
	Decimate	Mesh decimation
	Mesh Doctor	Repairs imperfections in mesh
	Move to origin	Move mesh to origin, to facilitate rotation of mesh when landmarking
	Mirror	Reflect specimen if desired side is damaged
Blender v2.79 (www.blender.org)	Various functions (e.g., Create Sphere, Sculpt)	3D mesh editing and creation of meshes to serve as the template
*Morpho* R package (Schlager 2017)	createAtlas	Creates an atlas from the template mesh, landmarks, curves, and surface points. For use in placePatch
	placePatch	The placement of surface points onto each specimen, using a template
	relaxLM	Sliding of semilandmarks to minimize bending energy or Procrustes distance across a dataset using the template as a reference
	slider3d	Sliding of semilandmarks to minimize bending energy or Procrustes distance across a dataset using the Procrustes consensus as a reference
	checkLM	Check correct placement of landmarks and sliding semilandmarks on meshes
*geomorph* R package (Adams and Otárola-Castillo 2013)	findMeanSpec	Identify specimen closest to the mean
	mshape	Estimate the mean shape for a set of aligned specimens
*shapes* R package (Dryden 2017)	shapes3d	Visualize landmarks and semilandmarks
*rgl* R package (Adler et al. 2018)	shade3d	Visualize mesh
	texts3d	Visualize the numbers of each landmark and semilandmark in the correct positions for each specimen. Used to identify erroneously placed semilandmarks
*LaMBDA* R package (Watanabe 2018)	lasec	Assess whether sufficient number of landmarks have been sampled to characterize shape variation
*paleomorph* R package (Lucas and Goswami 2017)	mirrorfill	Fill missing symmetrical landmarks
*Rvcg* R package (Schlager 2017)	vcgImport vcgPlyWrite	Mesh file format conversion

**Table 2:** Definitions for the terms used in this guide

Term	Definition
Landmark	Discrete point, ideally representing a biologically homologous position on a structure.
Curve	A series of sliding semilandmarks constrained to a defined outline, starting and ending at landmarks.
Curve point	A single sliding semilandmark on a curve.
Surface point	A single semilandmark placed on the surface of a structure defined by landmarks and curves.
Meshes	Three-dimensional reconstructions of specimens from CT scans and surface scans, typically stored in PLY or STL format.
Template	A surface mesh with landmarks, curves, and densely sampled single points within anatomical regions that is used to place surface semilandmarks on meshes of specimens.
Patching success	The placement of surface points onto a defined region, in the desired manner (e.g., achieving an even distribution of surface points, an absence of points falling outside the desired region, and an absence of points falling onto the incorrect side of the material).

**Fig. 4:**
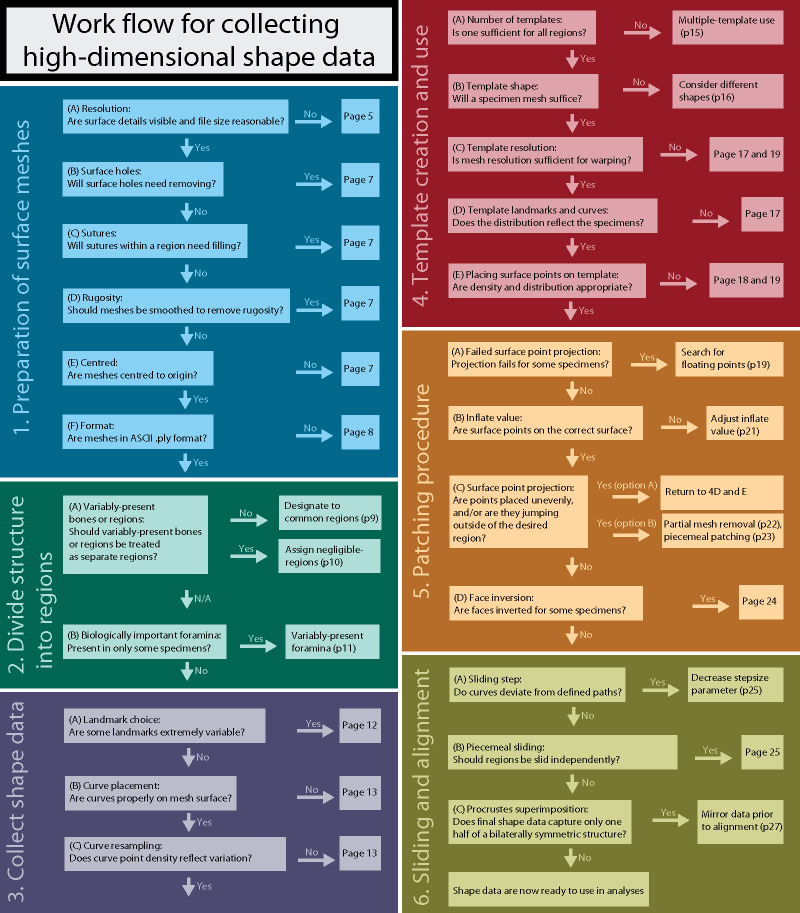
Suggested work flow for collecting high-dimensional shape data, summarizing the main steps and challenges that may arise during this process.

Effective application of this semi-automated patching procedure requires coordination of many interdependent steps, each with their own discussion points and potential pitfalls. These include (1) the selection and preparation of 3D meshes for the specimens and template, (2) designing a landmark scheme, and (3) implementing the patching procedure, sliding of semilandmark points, and Procrustes alignment. Here, we provide guidance for each of these steps and solutions to common issues. For a suggested work flow, see Fig. 4.

## Example datasets

We use empirical datasets to illustrate the requirements and recommendations for collecting high-dimensional data. These include three intergeneric studies sampling a wide range of diversity across archosaurs with 352 extant bird species (Felice and Goswami 2018), squamates with 181 species (Watanabe et al., 2019), caecilians with 35 extant species (Bardua et al. 2019), as well as frogs and salamanders. Many of the surface meshes used in these studies are available on phenome10k.org.

## Preparation of surface meshes

### Surface mesh resolution

The optimal surface mesh resolution (i.e., number of polygons) depends on the amount of variation present in the dataset and the aim of the study. The resolution should retain the geometrical features of the original structure, while not impeding the memory load (Souter et al. 2010). We found that surface meshes >∼50 Mb in size would significantly slow down Landmark Editor (although this is less of an issue if using Stratovan Checkpoint). For our intergeneric study of caecilian crania, surface meshes were simplified to ∼700,000 polygons (Bardua et al. 2019), and our frog dataset has a range of ∼200,000–2,000,000 polygons depending on the complexity of the mesh (since ornamented surface requires a higher number of polygons). Landmark-based morphometric studies will require resolutions sufficient for observing sutures, and high dimensional methods sampling entire surfaces will benefit from adequate surface detail being captured. Intraspecific datasets will typically require higher resolutions than interspecific datasets, as the former tend to exhibit smaller scale variation. Subtle differences between specimens in an intraspecific dataset may not be detected with decreasing resolution and will be more affected by digitization error. In contrast, much of the variation will still be detected with poorer resolution scans for datasets exhibiting relatively large variation. In a study comparing low-resolution surface scans to high-resolution CT scans, it was found that low-resolution was adequate for capturing variation in interspecific studies, whereas high-resolution was required for studies of asymmetry, as smaller biological signal can be heavily masked by noise (Marcy et al. 2018). Surface meshes can be decimated to an appropriate number of polygons using the “decimate” tool in Geomagic Wrap (3D Systems, Rock Hill) or the “Quadric Edge Collapse Decimation” tool in Meshlab (Cignoni et al. 2008) (Fig. 4, cell 1A).

### Fill surface holes

**Fig. 5:**
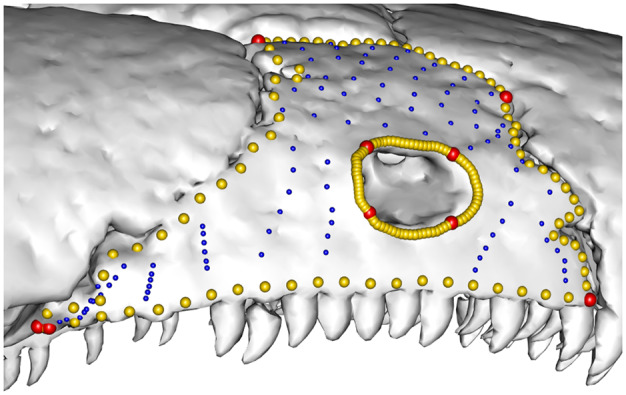
Fenestrae or large foramina can be excluded from a region by placing landmarks and curves around them, to prevent surface points sliding inside. Here, the orbit is excluded from the maxillopalatine region of *Gymnopis multiplicata* BMNH 1907.10.9.10 (viewed in lateral aspect). BMNH, Natural History Museum, London, UK.

**Fig. 6:**
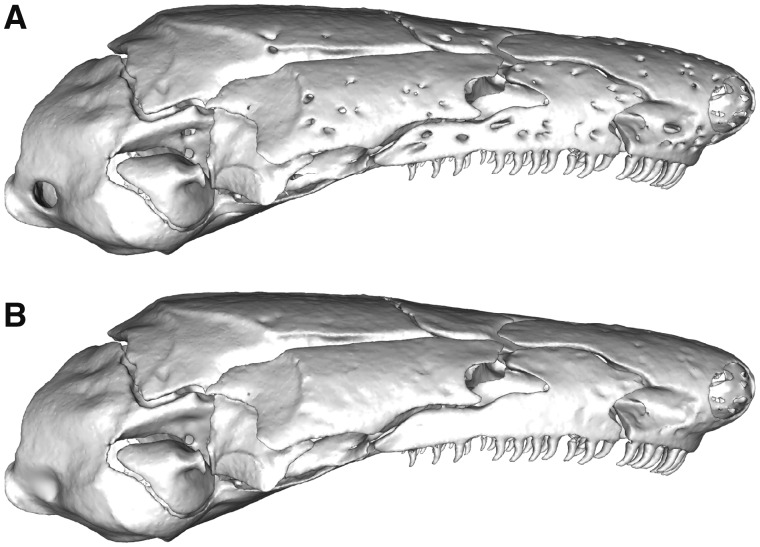
Removing foramina from surface meshes. *Idiocranium russeli* BMNH 1946.9.5.80, lateral view, before (**A**) and after (**B**) processing in Geomagic Wrap to remove the neurovascular foramina. BMNH, Natural History Museum, London, UK.

Each region onto which surface points are placed should largely be one continuous surface. Surface points can fall through holes during the patching procedure, so large foramina should be excluded from regions by placing curves to “fence off” these areas (e.g., the orbit within the maxillopalatine bone of some caecilians, Fig. 5). However, this is impractical when a specimen has many small, naturally occurring surface holes. Skulls can be' textured by numerous blind pits and neurovascular foramina, which vary in number and position across the clade. Small foramina such as these can be manually filled on the cranial reconstructions using Geomagic Wrap, providing this procedure does not alter gross morphology (Fig. 6). The decision to manually fill foramina should be based on the biological importance of the foramina for the research question (Fig. 4, cell 1B).

### Fill sutures within a region

**Fig. 7:**
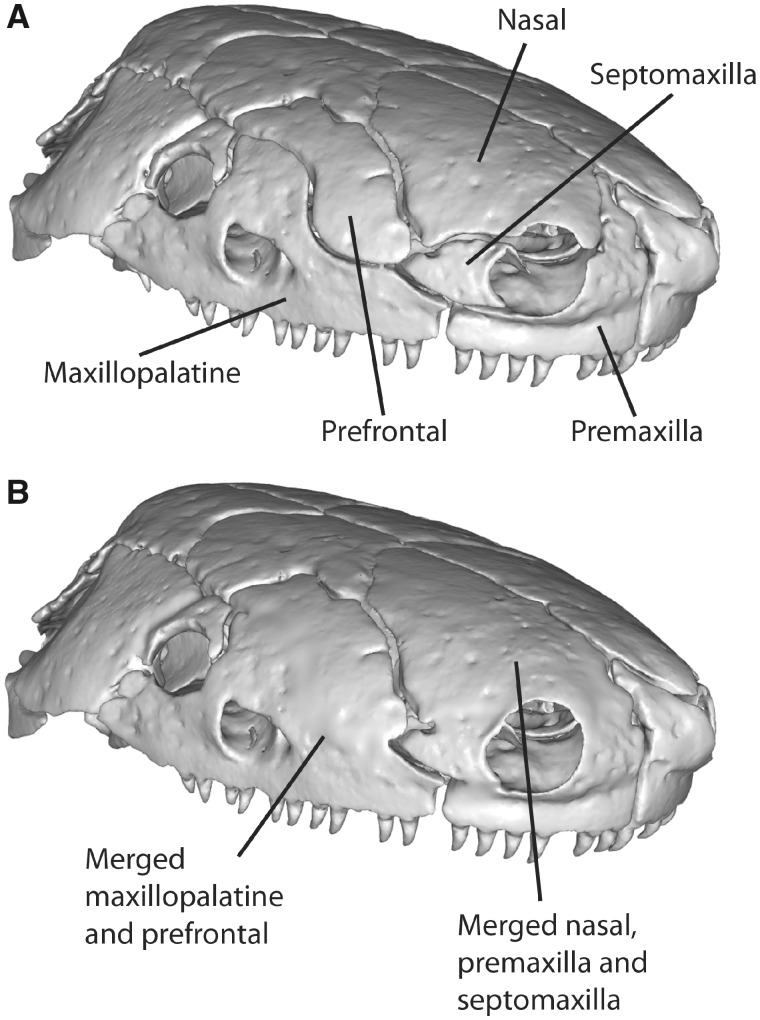
Removing sutures between adjacent bones. *Ichthyophis bombayensis* BMNH 88.6.11.1, dorsolateral view, before (**A**) and after (**B**) processing in Geomagic Wrap to remove the sutures between the maxillopalatine and prefrontal, and between the nasal, septomaxilla, and premaxilla. BMNH, Natural History Museum, London, UK.

Whereas many adjacent cranial bones are fused in clades such as Aves, bones are sometimes separated by unossified tissue, resulting in non-continuous surfaces across a structure in skeletal reconstructions based on standard CT scans. An example of this is the caecilian skull; most specimens have at least some individual cranial elements separated by unossified tissue. These gaps prohibit the patching of several bones as one region because they do not represent a continuous surface. Consequently, it may be necessary to fill in these gaps manually using Geomagic Wrap for bones constituting a single region. For caecilians, the prefrontal bone exists as a separate ossification to the maxillopalatine in only a few species. Therefore, the gap between these two bones was manually filled so that they can be patched as one region. In addition, the nasal, premaxilla, and septomaxilla variably fuse to form the nasopremaxilla, so that separate ossifications are manually merged into one continuous surface (Fig. 7) (Fig. 4, cell 1C).

### Rugosity

**Fig. 8:**
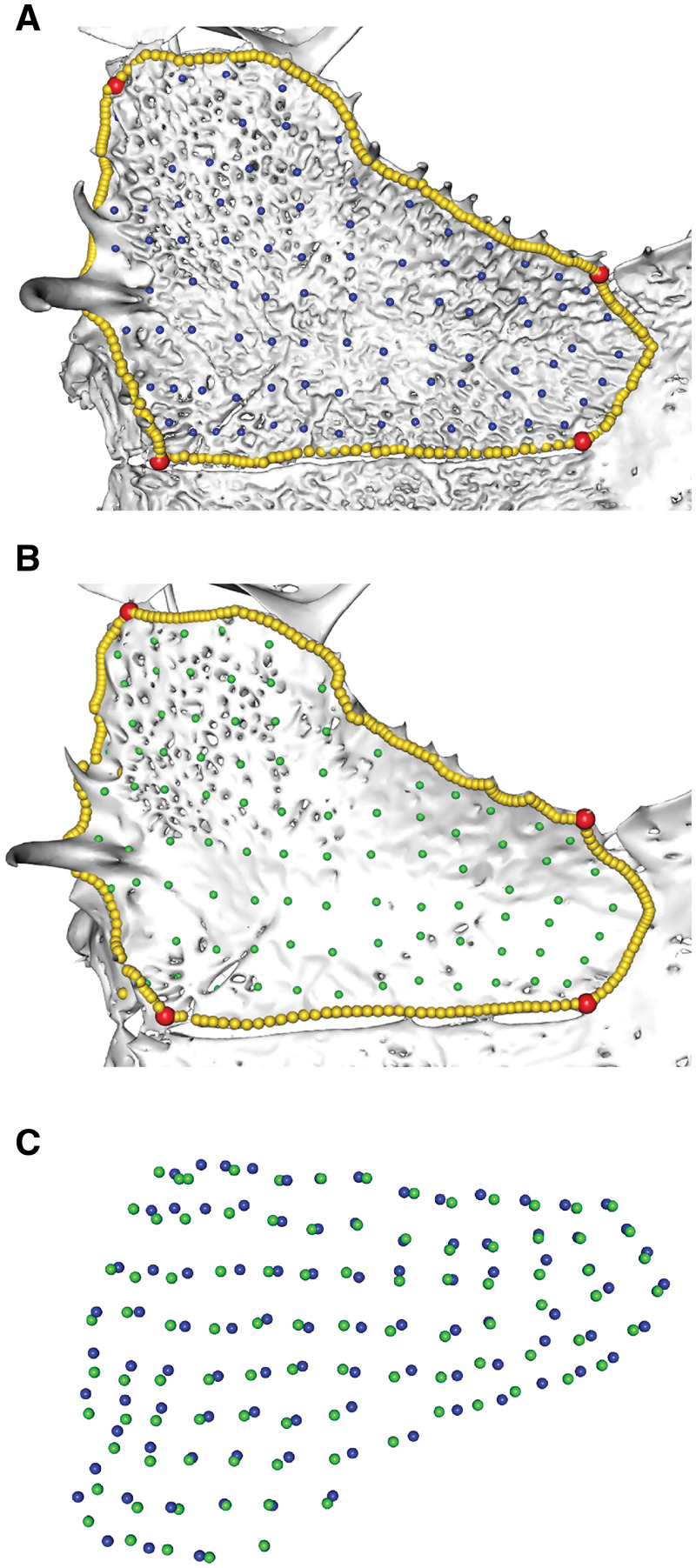
Effect of rugosity on patching. The frontoparietal of the frog *Anotheca spinosa* UF 137287, dorsal view, (**A**) with rugosity retained and (**B**) rugosity removed through use of the “remove spikes” function in Geomagic Wrap. (**C**) This density of surface points did not capture the rugose morphology, as surface points from the smoothed (green) and non-smoothed (blue) bones appear similar in distribution. Removing rugosity makes surface holes easier to identify, which can affect patching. UF, University of Florida, Gainesville, FL, USA.

Bone surfaces may be heavily rugosed or ornamented. These structures can be smoothed to remove or decrease rugosity if desired, using the “remove spikes” tool in Geomagic Wrap. We found that, for extremely rugose surfaces, removing rugosity facilitates the detection of foramina and the visualization of patching success. Our comparison of a surface patched with and without its rugosity (Fig. 8) demonstrates very similar results, despite the mesh surfaces looking different. We found that rugosity may only be represented by surface depth (by points landing on peaks and in troughs), as the density of surface points in a region will often be too coarse to accurately represent the high complexity of the surface. Overall, removing rugosity does not appear to greatly impact the capturing of overall shape when the density of surface points is coarser than the rugosity (especially when capturing shape over a disparate dataset). However, if rugosity is of specific interest, we suggest a high density of surface points to capture this complex surface. Semilandmarks have been shown to be capable of capturing ornamentation if desired, and they outperformed landmark data and outline data (elliptical Fourier analysis, see Giardina and Kuhl 1977; Kuhl and Giardina 1982) for capturing the shape of ornamented gastropod shells (Van Bocxlaer and Schultheiss 2010) (Fig. 4, cell 1D).

### Centre each surface mesh

Each surface mesh should be centered, to facilitate the rotation of the mesh when placing landmarks and curves in Landmark Editor (or Checkpoint Stratovan). This can be done using the “move to origin” function in Geomagic Wrap, or in the “Transform: Move, Rotate, Center” dialog box of Meshlab (Fig. 4, cell 1E).

### Format of surface meshes

Most meshes created from surface renderings of CT or surface scans are stored in Stanford Polygon Format (PLY) or Stereolithography (STL) format. Landmark Editor, as well as our analyses in R, requires meshes to be in PLY format. Specifically, the PLY files must be in American Standard Code for Information Interchange (ASCII), not binary format, for subsequent steps in R. To convert from STL or binary PLY to ASCII PLY, it is possible to import meshes into R using the function “vcgImport” from the R package *Rvcg* (Schlager 2017), and then export them using the function “vcgPlyWrite” from the *Rvcg* R package, specifying “binary=FALSE.” A common cause for the patching step failing to run is that meshes are stored as binary PLY files, not ASCII PLY files (Fig. 4, cell 1F).

## Dividing a structure into regions

### Overview

Dividing a structure into regions allows us to examine variation in potentially independent elements or modules and to investigate differential or localized influences on morphology such as allometry and ecological factors. However, the variable presence and fusion of bones within a dataset complicate the division of a structure into regions, as specimens must all have the same regions defined across the structure of interest if analyses under a unified framework are to be run. There are two options for bones that are variably present or variably fused across the sample (assuming we do not exclude them from the dataset altogether, which would create gaps in the physical representation of the structure). First, the bones could be placed into regions that are globally present across the dataset, based on shared development or function. Alternatively, they could be defined as individual regions, so that specimens lacking a region are designated an artificial “missing” region of negligible size (see below). Another complication is dealing with highly disparate regions. To define such a region, it may be necessary to use different landmarks and curves for subsets of specimens and use different templates to patch this region separately for each landmark and curve configuration. In this case, landmarks and curves can be removed after patching and only the surface points are retained for analyses, as the landmarks and curves would not be comparable across all specimens.

### Variably-present bones

#### Designate to common regions

**Fig. 9:**
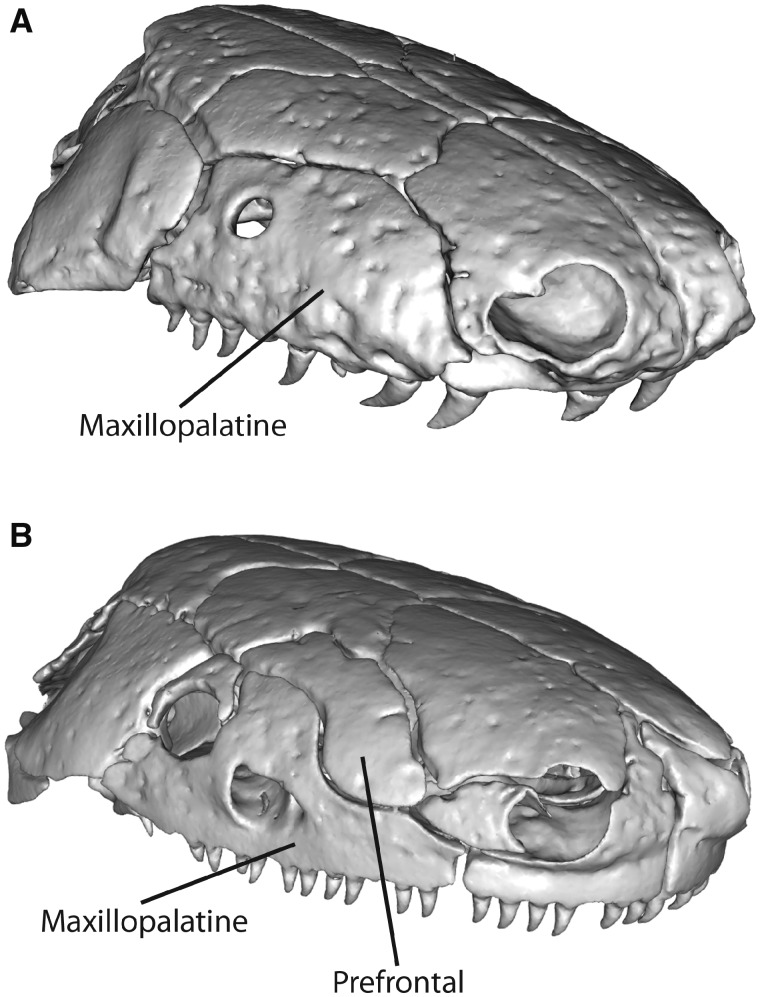
Variably present bones designated into regions present in all sampled specimens. (**A**) The maxillopalatine of *Caecilia tentaculata* BMNH field tag MW3945 (and most specimens of caecilians) is defined as one cranial region (Bardua et al. 2019). (**B**) The prefrontal of *Ichthyophis bombayensis* BMNH 88.6.11.1 is placed into the maxillopalatine region. These two regions are merged in Geomagic Wrap so that they are one continuous surface (see Fig. 7) Specimens in anterolateral view. BMNH, Natural History Museum, London, UK.

Variably-present or variably-fused bones (or regions) can be designated to regions globally present across all specimens. We recommend this procedure when there is a clear understanding of shared development or function, so that the merging is biologically informed. For example, the prefrontal bone in caecilians exists as a separate ossification in only some species, and thus it must be put into a region common to all caecilians. We place the prefrontal into a “midface” region along with the maxillopalatine (Fig. 9), as these two bones fuse in some species through development (Wake and Hanken 1982; Müller et al. 2005). Therefore, this region exists as the prefrontal and maxillopalatine for some species, and just the maxillopalatine for other species. Additionally, the nasal, premaxilla, and septomaxilla of caecilians can be placed into one “rostrum” region, as these all variably fuse to form the nasopremaxilla in some species. Thus, the rostrum region can be represented by one, two, or three separate ossifications (Fig. 4, cell 2A).

#### Assign negligible regions

**Fig. 10:**
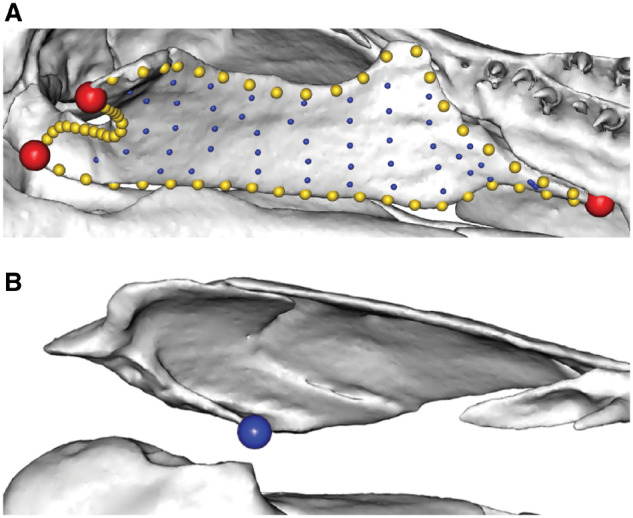
Negligible region method. The pterygoid region in two specimens, in ventral aspect: (**A**) *Epicrionops bicolor* BMNH 78.1.25.48 and (**B**) *Scolecomorphus kirkii* BMNH 2005.1388. The negligible pterygoid region of *S. kirki* is represented by the same number of surface points (blue), all occupying the same position. The area for this negligible region is therefore zero, or near zero, but it retains positional information. The position represents the likely location where this region would have been, if present. Landmarks (red points) and curves (yellow points) are removed before analyses for specimens with a present pterygoid region. BMNH, Natural History Museum, London, UK.

It may not always be reasonable to combine bones into one region, if there is no shared developmental or functional basis. Furthermore, it may not be suitable if doing so would greatly simplify or condense major regions or if the elements in question are absent in only a small number of specimens. In these cases, we apply a geometric morphometric approach previously suggested for studying novel structures (see Fig. 1b from Klingenberg 2008). If a variably-present bone is critical to characterize as a distinct region, it can be quantified as having “negligible” area when absent in some specimens (see Fig. 10). For example, within Gymnophiona, not all species have a functional pterygoid region which was defined as the pterygoid and/or the pterygoid process of the quadrate (Bardua et al. 2019). First, for specimens possessing this region, landmarks, curve points and surface points are applied as normal. For specimens lacking this region, a position is determined on the structure which best represents the location of the missing region, for example, a proximal position on an adjacent bone. The coordinates of this position are then replicated to achieve an array of *n* dimensions, where *n* represents the number of surface points characterizing this region when present in other specimens. Because we wish to define this region as zero size, we simply replicate the one position coordinate and use this as raw coordinate data, instead of applying the patching procedure for these specimens. Because this negligible region is not represented by landmarks and curves (only surface points), landmarks and curves used to define this region when present on other specimens are removed after the patching and sliding of the surface points for these specimens. This region is therefore only represented by surface points for analyses. Global Procrustes alignment will slightly adjust surface point positions such that the “negligibly sized region” is no longer zero size, but it remains near-zero in size and is still considered “negligible.” Although one could argue for exclusion of these variably-present structures, that approach would greatly limit the elements that could be considered in large-scale cross-taxon analyses and would result in inaccurate representation of the real biological variation in the sample of interest (Fig. 4, cell 2A).

### Biological foramina variably present: Negligible hole method

As mentioned above, the patching procedure requires surfaces to be a largely continuous surface, so biologically important holes, including the orbit and nares, must be “fenced” off with curves. Problems arise when only some specimens in the dataset have a fossa or foramen in the region to be patched. In these cases, specimens lacking a hole can be given a “negligibly-sized hole,” using the same landmarks and curves to fence off a miniscule area. This hole is approximately the size of one surface point, and our tests demonstrate that it does not affect patching (i.e., it does not create an empty space where the “negligibly-sized hole” was placed). This approach allows all specimens to be patched together as they all have the same landmark and curve configuration. The non-comparable landmarks and curves can then be removed before analyses (including Procrustes alignment).

**Fig. 11:**
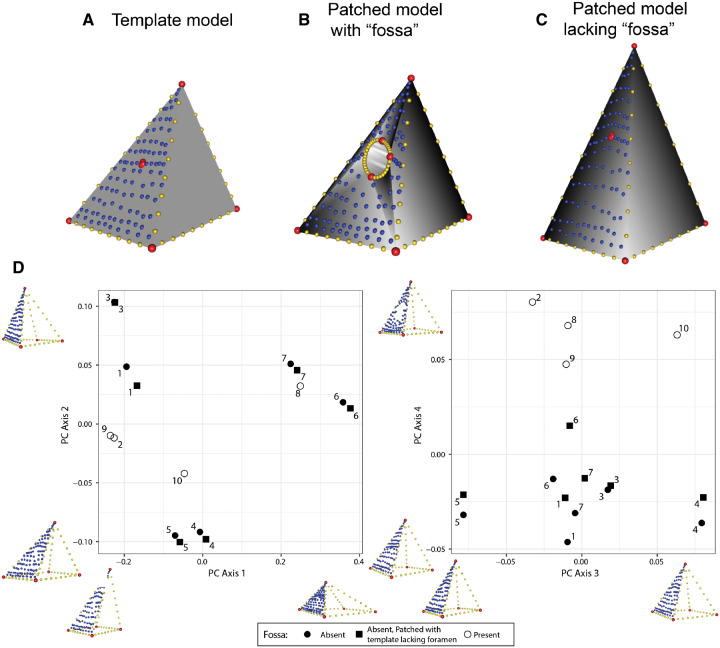
“Negligibly-sized hole” method for patching surfaces with variably present features. (**A**) Landmarks (red), curves (yellow), and surface points (blue) are digitized on a template mesh. (**B**, **C**) Surface points are projected on to target meshes. On meshes with “fossa,” curves are placed around the perimeter of this region. On specimens lacking the “fossa,” corresponding landmarks are placed extremely close together, forming a “hole” of negligible size (**C**). We subjected these data to a Procrustes alignment and principal component analysis (**D**). When that same specimen (numbered points) is patched with (solid circle) and without (solid square) the negligibly sized region corresponding to the “fossa,” these specimens share adjacent positions in morphospace.

For comparing across specimens with and without fossae, one should ensure that surface point placement is not appreciably affected by the presence of the “negligible” hole. To demonstrate, we tested patching with and without a negligibly-sized hole on 10 pyramidal 3D models of varying proportions using Blender v2.79 (www.blender.org). On 4 of the 10 models, we placed a circular “fossa” on one face (Fig. 11). An additional pyramidal model was produced to serve as a template mesh (Fig. 11A) (for more information regarding templates, please see the “Template creation and use” section). We placed landmarks on each vertex and curves along each edge. Landmarks and curves were digitized around the perimeter of the fossa (Fig. 11B) and corresponding curves were placed as a negligibly-sized hole on meshes lacking a fossa (Fig. 11C). On the template mesh, we digitized 90 surface points on a single face. Surface points were projected onto the 10 target specimens. The negligibly-sized hole technique allows surface points to be projected evenly on the surface of specimens lacking a fossa (Fig. 11C) and prevents surface points from being erroneously projected inside the fossa when present (Fig. 11B). We evaluated the effects of the negligibly-sized hole on the placement of surface points by repeating the patching procedure on the six pyramid meshes without fossae with the fossa landmarks and curves removed from the template and target meshes before patching. We then removed the fossa landmarks and curves from the original 10 specimen dataset and subjected all 16 specimens to a common Procrustes alignment and principal components analysis (PCA).

The first four principal component axes account for 96% of the cumulative shape variance in the dataset. The first principal component (PC1) describes the ratio of the base of the pyramid to its height, PC2 represents the angle of the face with surface points, PC3 is associated with variation in the angles of the corners of the base, and PC4 is correlated with the size of the fossa. Critically, pairs of identical pyramid shapes patched with and without the negligibly-sized hole share adjacent positions in morphospace (Fig 11D). This illustrates that this process for placing patches of surface points does not introduce undesirable artefacts in quantifying shape while also facilitating shapes with different anatomical features to be compared directly.

**Fig. 12:**
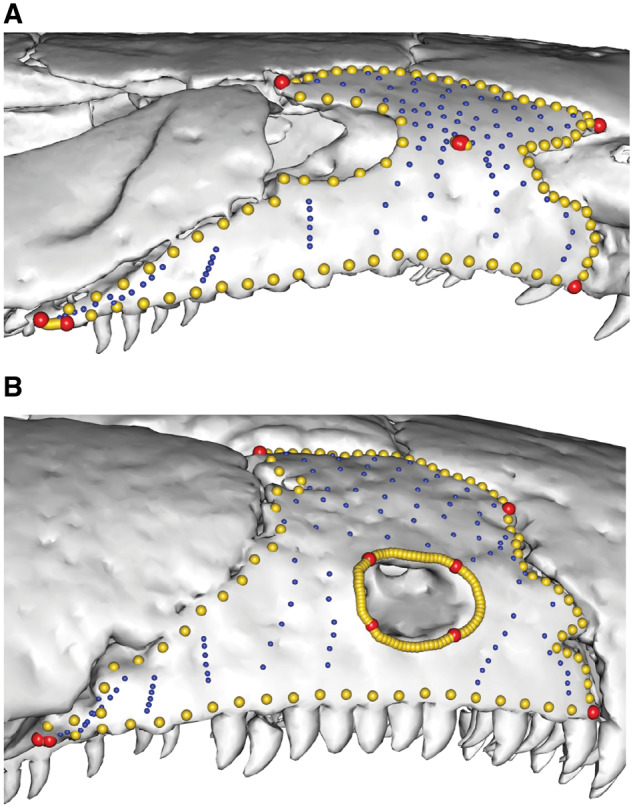
Negligible hole method for patching the maxillopalatine region of caecilians. (**A**) *Nectocaecilia petersii* BMNH 61.9.2.6 (no orbit or tentacular foramen completely closed in the maxillopalatine) and (**B**) *Gymnopis multiplicata* BMNH 1907.10.9.10 (orbit completely closed within the maxillopalatine). *Nectocaecilia petersii* had a “negligible hole” placed in the center of the maxillopalatine, so that these specimens could be patched together. Non-comparable landmarks and curves are then removed after patching. Specimens in lateral view. BMNH, British Museum of Natural History, London, UK.

A biological example of this situation occurs in the maxillopalatine of caecilians. This bone can have an orbit or tentacular foramen partially or completely enclosed within the bone. Complete enclosure of a foramen requires curves to “fence-off” this hole, whereas partial enclosure does not require a hole. However, to patch all specimens together, a negligibly-sized area was fenced off in the latter specimens, so that landmarks and curves were kept consistent (Fig. 12). One template can subsequently be used for these specimens (Fig. 4, cell 2B).

## Collection of shape data

### Landmark choice

**Fig. 13:**
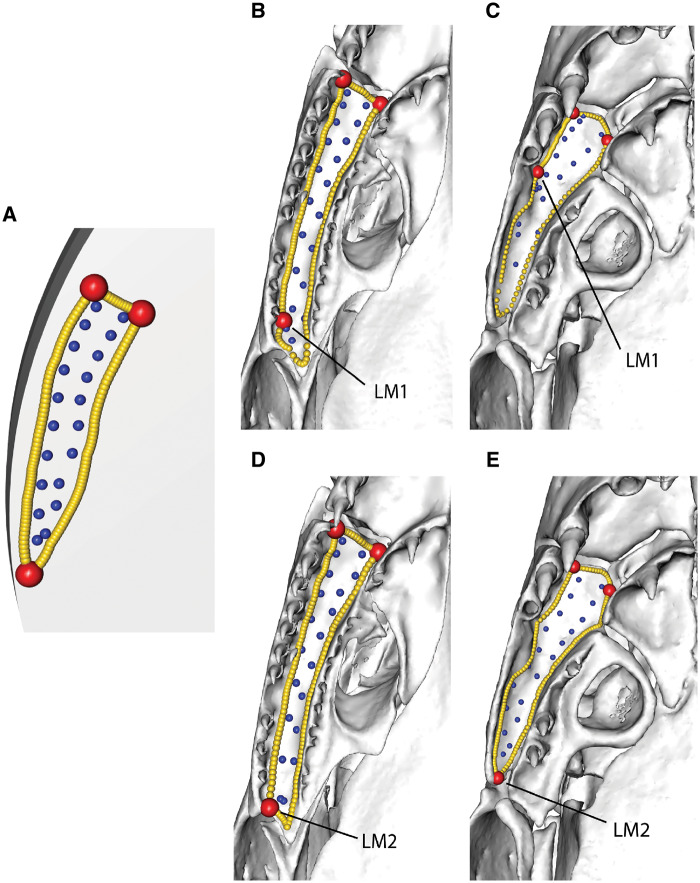
Landmark choice can affect patching success. Landmarks (red points) and curves (yellow points) are manually placed onto each specimen, and a template is used to semi-automatically place surface points (blue) onto each region. The success of this surface point placement can be affected by landmark choice. Here, a template (**A**) is used to patch the palatal surface of the maxillopalatine in (**B**, **D**) *Idiocranium russeli* BMNH 1946.9.5.80 and (**C**, **E**) *Luetkenotyphlus brasiliensis* BMNH 1930.4.4.1, using different landmarks (labelled “LM1” and “LM2”) to delimit the posterior extreme of this surface. (**B, C**) Landmark 1 (alveolus of ultimate tooth) may vary widely in position, making patching difficult, as the template can only resemble one morphology. (**D, E**) Landmark 2 (posterolateral extreme of the maxillopalatine) may improve patching success if they show less variation in landmark position, making the patching more successful. All specimens are viewed in ventral aspect, with anterior facing upwards. BMNH, Natural History Museum, London, UK.

Landmarks are divisible into three types, defined by biology (Type I), geometry (Type II), and relative positions (Type III) (Bookstein 1991), although Bookstein later redefined Type III landmarks as semilandmarks (Bookstein 1997). Type I landmarks are generally considered the most reliable and interpretable as they capture points with clear definitions, for example, tripartite sutures, but all three types are commonly used. The importance of landmark choice has already been discussed in detail, for example, for the human face (Katina et al. 2016) and in-depth discussions can be found in more general guides to geometric morphometrics (e.g., Bookstein 1991; Zelditch et al. 2004; Slice 2005). For certain structures, Type I landmarks may be difficult to identify, especially across a broad taxonomic scale. In this case, Type II landmarks may prove more useful both in terms of comparability and patching success. For example, in the caecilian dataset (Bardua et al. 2019), the landmark on the maxillopalatine defined by the “suture with the nasal and frontal” is not present in specimens possessing a prefrontal, as the prefrontal lies between these bones. However, a geometric landmark defined as the “anterodorsal extreme of the maxillopalatine” can be identified in all specimens. In addition, we find that an important consideration when determining landmarks for studies involving patching should be finding landmarks which do not vary widely in position across the sample. This is because surface point placement is the most successful when the landmark and curve configurations are similar across specimens. High variability in landmark position across specimens can make it difficult to find a template landmark distribution that will successfully place surface points onto every specimen. For example, a landmark defining the palatal surface of the caecilian maxillopalatine results in less variation in landmark position across specimens, which facilitates the placement of surface points (Fig. 13). Patching success is adversely affected by structures that are not strongly conserved in shape across specimens, so we advocate the use of landmarks which are the most conserved across specimens, in presence and position (Fig. 4, cell 3A).

### Curve semilandmark placement

It is important to ensure that the landmarks and curves accurately follow the outline of the desired region. When placing curve points in the IDAV Landmark Editor (or Stratovan Checkpoint) program, we recommend that they are placed on a flat surface, instead of on the sides of regions of interest. In other words, the normal of the landmarks and curve points should be consistent with the intended normal of the surface points. Although the normals of landmarks and curve points do not necessarily impact the placement of surface points, placing the anchoring curve points on the side may cause the additional curve points placed between these anchors by the program to be irregular in spacing. The extreme case is if the path between the anchored curve points deviates or falls from the perimeter of the region. This leads to incorrect placement of curve points (Fig. 4, cell 3B).

### Curve resampling

**Fig. 14:**
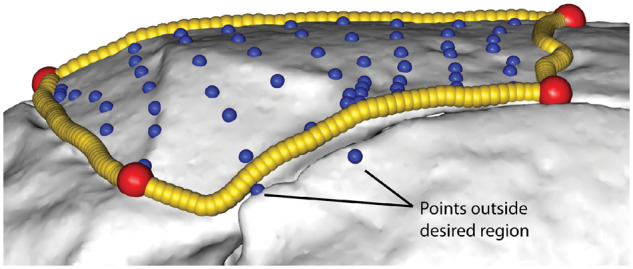
High-density curve points do not improve patching. A high density of curve points (yellow) placed on the parietal of the caecilian *Chikila fulleri* DU field tag SDB1304 does not prevent surface points (blue) being placed outside of the region of interest. Here, each of the four curves between the four landmarks was resampled to 50 points each, but two surface points were still not constrained to the desired region. Specimen view in lateral aspect. DU, Delhi University, New Delhi, India.

Because the placement of curve points on each specimen is done manually in Landmark Editor (or Stratovan Checkpoint), points are not usually evenly spaced along each curve, and the number of curve points initially chosen may not be ideally representative across the entire dataset. Curves are, therefore, resampled for even spacing before being slid during alignment (for code see SI in Botton-Divet et al. 2016). Sliding the curves after resampling is a crucial step, as equally spaced semilandmarks cannot be treated as optimally placed (see Fig. 1 from Gunz et al. 2005). For the caecilian dataset (Bardua et al. 2019), we tested how many points were optimal for resampling, by comparing over-representation of each curve (50 points per curve), under-representation (5 points per curve), and a vector of points which allocated more points to longer curves. We predicted that resampling curves to a high number of points would help constrain surface points to each region, as this leaves fewer “gaps” between adjacent semilandmarks through which points can “escape.” However, even with 50 curve points per curve, surface points can still fall outside of the region of interest (Fig. 14). In addition, having five points per curve did not adversely affect patching success compared to the oversampled scheme. Increasing the number of curve points actually seems to result in more specimens failing to patch (i.e., errors messages returned for these specimens) (see “placePatch” function). When the “relax.patch” argument is set as true (relax.patch=TRUE) in the “placePatch” function, patching success is considerably higher when curves are resampled to 5 points per curve (only one specimen failed to patch for our caecilian dataset of 35 specimens) instead of 50 (11 specimens failed to patch). This outcome suggests that oversampling of curve points can actually impede the patching process. Our recommendation is to resample the curves based on their original length, but in most cases to limit each curve to no more than ∼20–30 points. This level of sampling results in curves that are well represented in typical cases, without compromising patching. Furthermore, we recommend that the density of curve points is similar to the density of surface points to achieve even coverage of the structure (Fig. 4, cell 3C).

## Template creation and use

### Overview

While landmarks and curves are manually placed onto every specimen, the surface points are only placed onto one mesh, and these surface points are then projected onto each specimen from this one mesh (Schlager 2017). The one mesh onto which the surface points are placed is referred to as the “template,” and the success of the surface point projection onto all specimens is greatly dependent on the template’s resolution, shape, and distribution of landmarks, curves, and surface points. Previous studies have either placed the surface points onto the template manually (Watanabe et al., 2019; Fabre et al. 2013a, 2013b; Botton-Divet et al. 2016; Felice and Goswami 2018; Bardua et al. 2019; Marshall et al. 2019) or automatically (by generating a mesh of roughly equidistant points, Aristide et al. 2016), but we will limit discussion to the manual placement of surface points onto the template, to control where points are placed, and to control how many points are placed in each region. Surface points are placed onto the template in the same way that landmarks are (using the “single point” option in Landmark Editor or Checkpoint), and these are then considered surface points once loaded into R. The surface point projection is achieved using the landmarks and curves on each specimen as reference, as the template will have the same distribution of landmarks and curves. The template’s mesh, landmarks, curves, and surface points are all imported into R, and are used in the “createAtlas” function in the *Morpho* package to create an atlas, which is subsequently used in the patching step to project the surface points onto each specimen. Because the atlas is simply the association of the template’s mesh with the template’s landmarks, curves and surface points, we will continue to use the term template instead of atlas here.

### Number of templates

In certain taxonomic sampling, identical configurations of landmarks and curves in every region across all specimens may not be possible. In such cases, more than one template may be required for a region, because a single template can only patch specimens with identical landmark and curve configurations. Variable regions should be represented using as few landmark and curve configurations as possible. One template should be used to patch each region when possible, so that bending energy can then be minimized across all specimens in the subsequent sliding step. However, when more than one template is required for a region, specimens with regions that have each landmark and curve configuration are patched as groups. Landmarks and curves are removed if necessary (when these are not consistent across the dataset), and then the remaining landmarks and curves and the surface points from each variable region are added to the data collected for the globally present regions. When more than one template is used, the surface points are only slid as groups and not globally, so it is important to be careful about where the points are placed on the template. Surface points on different templates should be placed in analogous ways, so that the data are comparable. Once all coordinate data have been collated from all templates, Procrustes alignment is applied to the complete dataset prior to any further analyses.

**Fig. 15:**
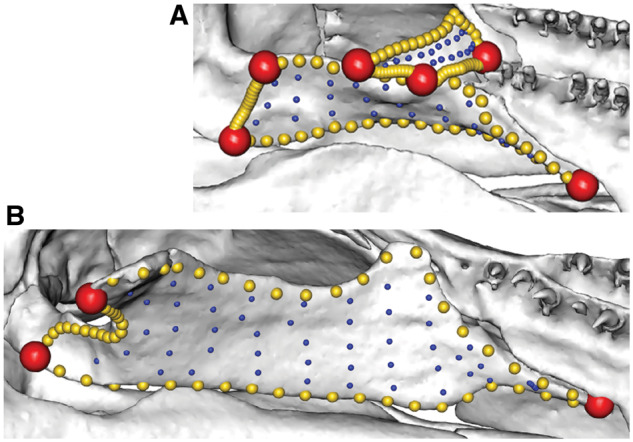
Multiple templates used for highly disparate regions. The pterygoid region as defined by this study, in ventral aspect, for *Praslinia cooperi* BMNH 1907.10.15.154 (**A**) and *E. bicolor* BMNH 78.1.25.48 (**B**). Because this region consists of either one (**B**) or two (**A**) bones, landmarks and curves are not consistent in number or position across specimens. Here, landmarks and curves are used to constrain the regions, and the pterygoid region is patched separately in specimens with one or two bones. Curves and landmarks are removed after patching, while keeping the surface points. BMNH, Natural History Museum, London, UK.

**Fig. 16:**
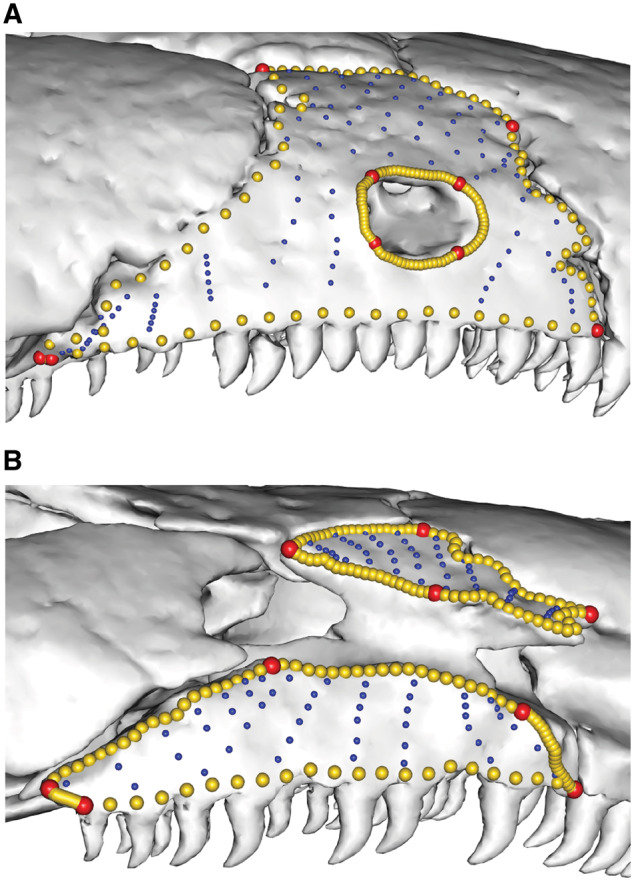
Multiple templates used for highly disparate regions. The maxillopalatine can have a tentacular foramen completely enclosed within the bone (*Gymnopis multiplicata* BMNH 1907.10.9.10, **A**), or a tentacular fossa passing through its entire length (*Chthonerpeton indistinctum* MCP field tag MW16, **B**) or neither. These require different patching approaches, but once patched, the curves and landmarks can be removed and the surface points analyzed. Specimens in lateral aspect. BMNH, British Museum of Natural History, London, UK; MCP, Museu de Ciências e Tecnologia da PUCRS, Porto Alegre, Brazil.

Caecilian crania are highly variable and require the use of multiple templates (Bardua et al. 2019; Marshall et al. 2019). As an example, the pterygoid region in caecilians was defined in our study to be the pterygoid process of the quadrate, and/or the pterygoid (ectopterygoid) when present. One template could not represent both variations, so specimens with one bone present were patched together, and specimens with both bones present were patched together (Fig. 15). The ordering and distribution of surface points were analogous across the two templates, with the posteriorly positioned surface points on the “single bone” specimens corresponding to the surface points placed on the posterior bone in the “two bones” specimens (and similarly with the anterior surface points). Pterygoid landmarks and curves were removed from the resulting datasets as these differed across the morphologies, so only the surface points were retained. Similarly, when the tentacular fossa runs the entire length of the maxillopalatine in caecilian crania, the maxillopalatine must be patched as two regions, dorsal and ventral to this fossa (Fig. 16). Specimens whose maxillopalatine has a tentacular foramen completely enclosed within the bone, however, are better represented by a template with one region and a hole. Surface points were placed on each of these two maxillopalatine templates such that the first half were dorsal to the tentacular fossa/foramen, and the second half were ventral, with analogous distributions (Fig. 4, cell 4A).

### Template shape

The most suitable template shape depends on the variation observed across the dataset. Previous studies have used a specimen from the dataset (e.g., Aristide et al. 2016; Botton-Divet et al. 2016; Marshall et al. 2019), a non-sample specimen (Wölfer et al. 2019), or a geometrically simplified representation of the structure under question (e.g., Fabre et al. 2014; Felice and Goswami 2018; Bardua et al. 2019). Intraspecific datasets typically exhibit smaller variation in morphology. As such, template shape which represents the actual morphology of the species will likely result in a successful placement of surface points (Souter et al. 2010; Marshall et al. 2019). The specimen closest to the average morphology can be determined through use of the “findMeanSpec” function in the *geomorph* R package. The surface mesh of this specimen can be used to create the template with the full configuration of landmarks and semilandmarks. Alternatively, a specimen can be picked at random to use as the template if the morphological variation is especially small. However, the use of a specimen as a template may not be appropriate for broad taxonomic studies because its morphology may not be generalizable across the entire breadth of shape variation. A study comparing the most suitable template shapes for two datasets found that the dataset exhibiting extreme morphological variation (theropod pelvic girdles) required a considerably geometrically simpler mesh than the dataset exhibiting only small morphological variation (shrew skulls) (Souter et al. 2010). No one specimen’s morphology in the theropod pelvic girdle dataset would have sufficiently represented the morphology captured across the entire dataset. It was found that the greater the morphological variation, the simpler the template should be. This is because the template is warped (see “Warping of template” section), so that while a specimen’s mesh will warp accurately to other specimens’ meshes when the morphologies are similar, this is more difficult when the morphologies are very different, as a complex shape has to transform into another complex shape (Souter et al. 2010). A simpler shape in this case will warp better to each specimen’s morphology. For our studies of caecilians, squamates, and birds, we found that a generic hemispherical mesh as the template was effective at placing patch semilandmarks (see “Warping of template” section). A hemisphere was more successful than a sphere with respect to accuracy in patching, as the former better represents the shape of a skull (with the ventral cranial surface as the flat surface of the hemisphere, and the tooth row following the base of the hemisphere). These template shapes can be created in programs including Meshlab and Blender (Fig. 4, cell 4B).

### Template resolution

**Fig. 17:**
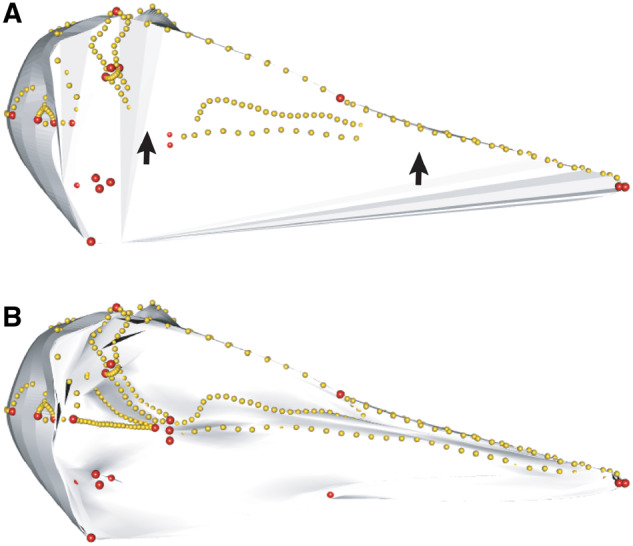
Warping template meshes of different resolutions. (**A**) Low polygon (1,802 faces) and (**B**) high polygon (18,024 faces) hemispherical meshes warped to the shape of the bird *Alca torda* (NHMUK 1897.2.25.1), ventral view. The warped low-resolution template is a poor fit with the landmark configuration of the target specimen, producing areas where the contours of the mesh do not correspond to the curves (black arrows). In contrast, the shape of the warped high-resolution mesh exhibits more detailed shape deformation and greater correspondence with target configuration. This improves the performance of the projection step of the patching method.

The resolution of the template mesh is equally as important as the template shape. Surface points are projected from the warped mesh onto the target specimen. Therefore, patching accuracy is partially dependent on how well the warped template mesh fits with the topology of the target mesh. It is essential that the template mesh has sufficiently high resolution (i.e., consists of enough triangles), so that the template can be warped to accurately reflect each specimen’s morphology. The number of polygons limits the degree to which the template mesh can be deformed (Fig. 17). Very low-resolution meshes thus produce poor correspondence between template and target specimens. The template must, therefore, have a high-resolution but does not have to resemble the specimen morphology. The necessary number of faces for the template mesh will vary based on the complexity of the morphology being quantified, but hemispherical templates with around 18,000 faces have proven suitable for vertebrate skulls (Fig. 4, cell 4C).

### Template landmarks and curves

How regions are defined on the template can impact patching success. For datasets with small amounts of variation, the landmark and curve positions on the template can follow a pattern based on the average shapes of each region in the target specimens. However, interspecific studies encounter considerably more variation in morphology. An inevitable result of studying shape variation across a diverse dataset is that extreme shapes and sizes form part of the dataset. The template’s landmarks and curves must, therefore, be suitable for these extreme shapes as well, and an average shape may not be the optimal solution. For regions exhibiting large size variation, we found the most success when the template represented the morphology of the smaller-sized regions. Surface points could successfully fill a large region on a specimen when the template represented a small shape, with densely clustered surface points, but issues arose when widely distributed points from the template were patched onto a small region. Surface points would often fall outside the desired region.

**Fig. 18:**
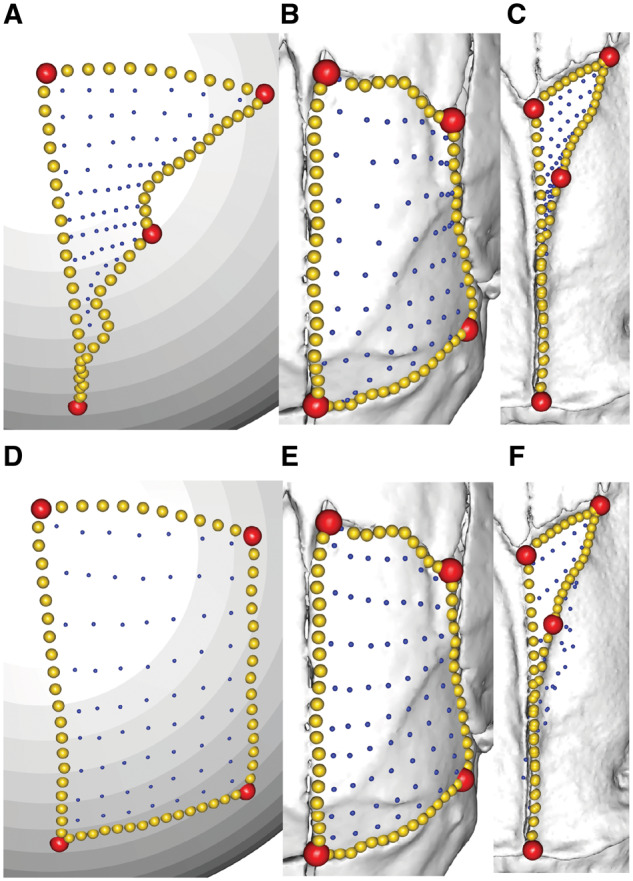
Effect of template shape on patching success. The external surface of the parietal is rectangular in shape for most caecilian species (seen here in dorsal aspect, with anterior facing upwards), but can appear more triangular in some species. To illustrate the effect of template shape on patching success, two templates were used to patch the parietal for two caecilian species. (**A**) A triangular-shaped template successfully patches the parietal of both (**B**) *Microcaecilia albiceps* MCZ A-58412, and (**C**) *R. bivittatum* BMNH field tag MW2395, whereas (**D**) a rectangular-shaped template patches (**E**) *M. albiceps* well but (**F**) *R. bivittatum* poorly (surface points have fallen outside the desired region). In this case, the most globally successful template was not the one resembling the most common morphology, but the one resembling the extreme morphology. BMNH, Natural History Museum, London, UK; MCZ, Museum of Comparative Zoology, Cambridge, MA, USA.

One example is the parietal of caecilians (Fig. 18). For the purposes of analyzing external bone surfaces, the adductor muscle ridge was taken as the lateral margin of the parietal when a squamosal-parietal fenestra is present. Whereas most taxa exhibit an approximately rectangular-shaped parietal, two species (*Rhinatrema bivittatum* and *Epicrionops bicolor*) have a more triangular-shaped external surface of the parietal. We found that a triangular-shaped template outperformed a rectangular-shaped parietal by keeping the surface points inside the desired region. Therefore, despite most specimens having a rectangular-shaped parietal, the template that was the most globally successful imitated the shape of the parietal in *R.bivittatum* and *E.bicolor*. A rectangular template resulted in posteriorly positioned points falling outside the parietal for *R.bivittatum*. Surprisingly, a triangular shaped template configuration for this region successfully patched every specimen. This suggests the patching procedure is more successful at enlarging the spaces between points, than at decreasing spaces between points (compare posterolateral points). Hence, the use of mean shape is not necessarily the most effective template for patching (Fig. 4, cell 4D).

### Number of surface points 

The optimum number of surface points to place onto the template depends on the complexity and the size of each defined region. More points may better represent a region, but we found this also increases the likelihood of some points falling outside the region of interest. In addition, over-representation of a region unnecessarily increases the dimensionality of the dataset, which could lessen power of the analyses that follow (for a discussion on the optimal number of landmarks/semilandmarks, see Watanabe 2018). For regions exhibiting large size variation, the number should be high enough to allow the largest region to be represented. For our interspecific cranial datasets, we used ∼500–1000 surface points to represent the entire cranium. Regions varied from having ∼12 to ∼100 surface points. The occipital condyle, for example, has a small and simple surface so was generally represented by ∼20 surface points, whereas the maxillopalatine is a large region and was represented by 48 surface points. The numbers of surface points are within the range of previous studies, which have used 24 surface points to capture the articular surface of the humerus (Fabre et al. 2014), 225 for musteloid crania (Dumont et al. 2015), 265 for the surface of the entire humerus of primates (Fabre et al. 2017), 268 for monkey endocasts (Aristide et al. 2016), 800 for shrew crania (Cornette et al. 2013), and <800 surface points for shrew mandibles (Cornette et al. 2013).

**Fig. 19:**
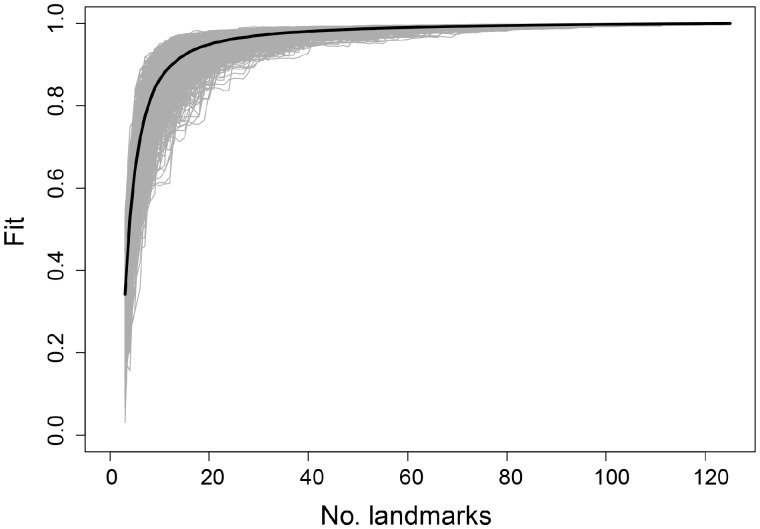
Sampling curve from performing LaSEC on the frontal region of the caecilian dataset. Each gray line indicates fit values from one iteration of subsampling. Thick, dark line denotes median fit value at each number of landmarks. The presence of a plateau indicates robust shape characterization.

At present, it is not possible to determine a priori how many surface points are necessary to fully capture the shape variation. However, it is possible to retrospectively examine how many (semi) landmarks are required to capture the shape of a region, through implementation of the “lasec” function in the R package *LaMDBA* (Watanabe 2018). This function subsamples the original dataset by randomly selecting 3, 4, 5, … *n* points, determining the fit of each reduced dataset to the complete dataset, and repeating this for a selected number of iterations. Fit is based on Procrustes distance between the full and subsampled datasets with respect to position of the specimens in high-dimensional morphospace (i.e., not the spatial position of the landmarks). We performed LaSEC for landmarks and semilandmarks (curve and surface points) for the caecilian and squamate datasets, for individual cranial regions. The function generates a sampling curve, where a plateau in the curve signifies stationarity in characterization of shape variation and absence of this plateau indicates inadequate characterization. The curves from each cranial region (e.g., Fig. 19) clearly show that enough landmarks and semilandmarks had been sampled due to a robust plateau in the curve.

We also determined the number of landmarks and semilandmarks that would have been sufficient for each region, given a required fit of 0.9, 0.95, and 0.99 between the reduced and complete datasets (Tables 3 and 4). These results could be used as a guide for estimating how many landmarks/semilandmarks should be taken for comparably sized regions. As a general guide for cranial regions, we suggest 12+ landmarks/semilandmarks for small and topologically simple regions (e.g., jaw joint articular surface), and ∼ 70 landmarks/semilandmarks for larger and morphologically complex regions (e.g., occipital region). Use of LaSEC revealed that we did capture shape accurately in all datasets, and that fewer landmarks/semilandmarks would have still captured shape in great detail. However, this cannot be determined in advance, and so we suggest it is preferable to oversample a structure and later downsample if necessary. We, therefore, suggest placing a relatively high density of surface points onto each region of the template, and then use LaSEC to guide downsampling if required. Because surface points should be placed evenly across structures, it is necessary to also consider which region may require the highest density of surface points (i.e., which region may be particularly complex and varying in morphology). If one region requires a high density of points to characterize shape, the remaining regions should have a similar density of surface points in order to ensure even coverage of the entire structure of interest (Fig. 4, cell 4E).

**Table 3:** Results from performing LaSEC with 1000 iterations on individual cranial partitions of the extant caecilian dataset.

Dataset	Number of landmarks	Total number of landmarks and semilandmarks	Fit = 0.90	Fit = 0.95	Fit = 0.99	Fit of landmark-only dataset
Basisphenoid region	4	155	15	25	69	0.583
Frontal	4	125	13	21	61	0.617
Jaw joint	3	50	13	19	37	0.306
Maxillopalatine (interdental shelf)	4	110	13	19	52	0.782
Maxillopalatine (lateral surface)	3	134	14	23	64	0.238
Maxillopalatine (palatal surface)	5	75	13	19	44	0.602
Nasopremaxilla (dorsal surface)	7	148	13	21	61	0.684
Nasopremaxilla (palatal surface)	3	59	8	12	29	0.770
Occipital condyle	2	34	11	15	27	NA (only two landmarks)
Occipital region	5	153	16	27	73	0.605
Parietal	3	126	11	18	51	0.361
Pterygoid	0	50	7	10	24	NA
Quadrate (lateral surface)	2	57	12	18	38	NA (only two landmarks)
Squamosal	4	104	15	25	61	0.574
Stapes	0	20	10	12	17	NA
Vomer	3	69	12	18	41	0.538
Total						

“Number of landmarks” lists the number of fixed landmarks in each region. The columns “Fit = 0.90,” “Fit = 0.95,” and “Fit = 0.99” indicate the median number of subsets of landmarks needed to achieve the fit between the subsampled and full datasets. Fit values are based on Procrustes sum of squares between the subsampled and full datsets ranging from 0 to 1 denoting poor to perfect correspondence between the distributions of shape variation, respectively. The column “Fit of landmark-only dataset” indicates the fit value between the landmark-only dataset and landmark plus semilandmark dataset for each region. For definitions of cranial regions, see Bardua et al. (2019).

**Table 4:** Results from performing LaSEC with 1000 iterations on individual cranial partitions of the extant squamate dataset

Dataset	Number of landmarks	Total number of landmarks and semilandmarks	Fit = 0.90	Fit = 0.95	Fit = 0.99	Fixed-only
Premaxilla	4	78	15	23	49	0.713
Nasal	4	86	15	25	54	0.664
Maxilla	5	162	16	27	74	0.696
Jugal	3	94	13	20	51	0.645
Frontal	4	130	14	25	66	0.721
Parietal	4	98	16	28	64	0.647
Squamosal	3	52	17	25	43	0.452
Jaw joint	4	42	20	27	38	0.484
Supraoccipital	5	132	30	55	90	0.597
Occipital condyle	2	37	22	27	34	NA
Basioccipital	4	122	14	26	66	0.805
Pterygoid	3	53	14	21	39	0.421
Palatine	4	64	16	23	45	0.457

“Number of landmarks” lists the number of fixed landmarks in each region. The columns “Fit = 0.90,” “Fit = 0.95,” and “Fit = 0.99" indicate the median number of subsets of landmarks needed to achieve the fit between the subsampled and full datasets. Fit values are based on Procrustes sum of squares between the subsampled and full datsets ranging from 0 to 1 denoting poor to perfect correspondence between the distributions of shape variation, respectively. The column “Fit of landmark-only dataset” indicates the fit value between the landmark-only dataset and landmark plus semilandmark dataset for each region. For details on the cranial regions see Watanabe et al. (2019).

### Surface point distribution

The distribution of surface points placed on the template will depend on the shape variation of each region, across all specimens. Considering the most appropriate distribution for each region is crucial, as this can strongly affect the patching process. Where possible, we recommend a systematic distribution, consisting of rows of evenly spaced surface points parallel to the curves defining each region. One should place surface points away from curve points, to reduce the risk of points falling outside the desired region. Use of more than one template requires additional consideration, as surface points from corresponding regions should always be equivalent in position (Fig. 4, cell 4E)

### Warping of template

**Fig. 20:**
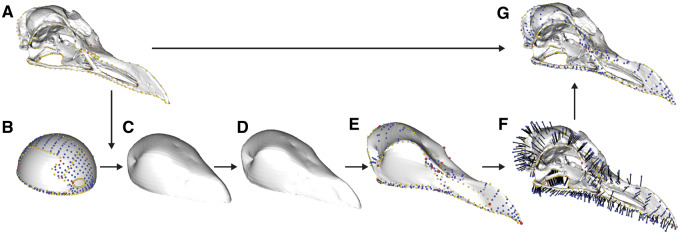
Projecting patch points from template to specimen. Morphology of a specimen (**A**, *Alca torda*, NHMUK 1897.2.25.1) is quantified coarsely with 3D landmark data (red: anatomical landmarks, yellow: curve points). Corresponding landmarks and curves are digitized on a template mesh, along with high density surface points (blue) which will be transferred from the template to the target specimen (**B**). The template is then morphed to the shape of the specimen (**C**, **D**), generating the intermediate model with surface points (**E**). Surface points are projected from the intermediate model on to the specimen (**F**), producing dense representation of entire surface of interest. NHMUK, Natural History Museum, London, UK.

The patching procedure implemented in the R package *Morpho* semi-automatically projects surface points on to a target mesh (Fig. 20A) from a template mesh upon which surface landmarks have been digitized (Fig. 20B). An essential part of this process is the warping of the template mesh via TPS deformation based on the curves shared between the template and target specimens (Fig. 20B–E and Video 1). To prevent the surface points from being misplaced within the target mesh, the surface points are “inflated” along their normals (Video 2) and then projected back until each landmark contacts the target mesh (Video 3) (Fig. 4, cell 4C).

## Patching procedure

### Failed surface point projection

**Fig. 21:**
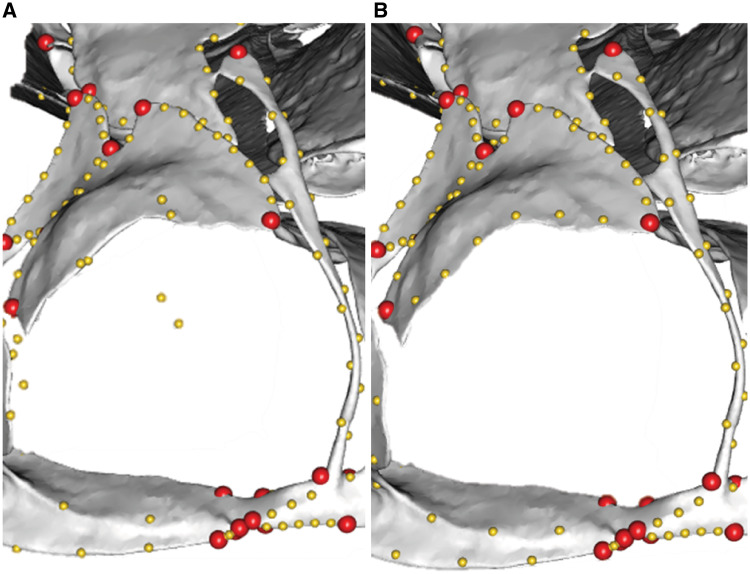
Incorrect and correct placement of curves (yellow points) on a salamander, *Bolitoglossa adspersa* ZMB 71710. (**A**) One curve has been defined incorrectly, so some curve points have fallen off of the bone. The floating curve points may then result in the patching step failing. (**B**) When correctly defined, this curve traces the anterior margin of the nasal. Specimen in anterior aspect. ZMB, Zoological Museum of Berlin, Berlin, Germany.

The patching of some specimens in a dataset can fail (i.e., an error message is returned). This can happen both when the relax.patch argument is set to TRUE or to FALSE when running the “placePatch” function in *Morpho*. This specifies whether to minimize bending energy toward the atlas (the template). We found the likelihood of specimens failing to patch was increased when relax.patch was set to TRUE. The specimens that fail often have some curve points that are “floating” and are not completely sitting on the surface of the mesh (Fig. 21). However, these “floating” points can be difficult to notice as they can be just above the mesh surface. This can be a consequence of curves being defined incorrectly, or curves being placed too near the edge of a bone and then sliding off the surface. For specimens whose patching fails, we recommend checking the placement of the curve points carefully (Fig. 4, cell 5A).

### Inflate value 

**Fig. 22:**
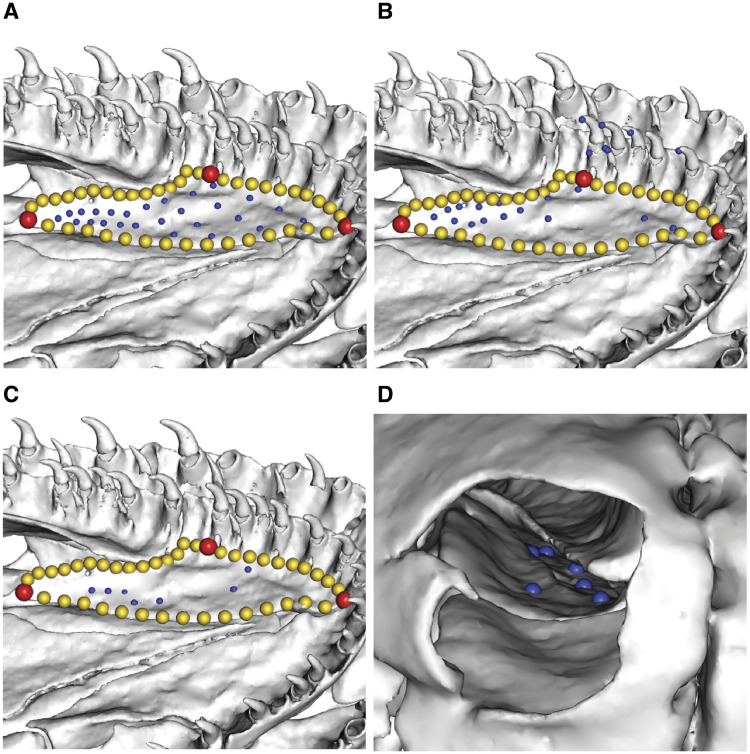
The effect of adjusting the inflate value (*I*) during the patching step, using the vomer of the caecilian *E. bicolor* BMNH 78.1.25.48 as an example. (**A**) Here, all surface points have been correctly placed onto the outer surface of the vomer (*I *= 0). (**B**) When the inflate value is too high, surface points can jump outside the region of interest, onto nearby material (teeth in this case) (*I *=* *1). (**C**) When the inflate value is too low, surface points can be placed on the internal surface, as seen through the nares in (**D**) where the points are now on the underside of the vomer (*I* = −0.1). Specimen displayed in (**A–C**) ventromedial and (**D**) anterior aspect. Ideal inflate values will vary for each region, and possibly each specimen. BMNH, Natural History Museum, London, UK.

During patching, it is possible that points are not always placed onto the external bone surface, especially if the bone material is thin. Points may instead fall onto the internal bone surface. This can be corrected by increasing the inflate value of the “placePatch” function in *Morpho* (Fig. 22).

Conversely, if the inflate value is too high, it is possible for surface points to fall outside of the region of interest, with surface points projected onto nearby surfaces outside of the defined region (Fig. 22). This appears to pose the greatest problem when the desired region is in close proximity to another surface. When patching the palate, nearby teeth are especially problematic as their surface is often nearby and parallel to the normal vector of the surface points. This issue can normally be fixed by reducing the inflate value just for the palate, although teeth may have to be removed if an optimal inflate value cannot be found that places the surface points neither on the internal bone surface nor on nearby surfaces.

**Fig. 23:**
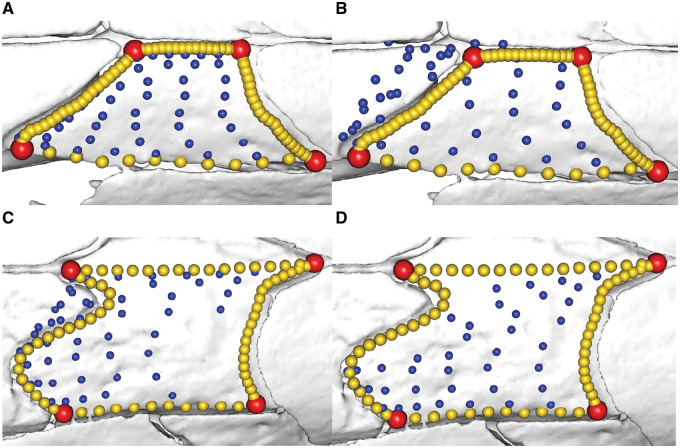
Optimal inflate value can vary across specimens for the patching step. The optimal inflate value (*I*) may vary across the dataset, as can be seen for the frontal bone across caecilians. (**A**) and (**C**) show the patching result using *I *=* *0 for *Scolecomorphus kirkii* BMNH 2005.1388 and *Schistometopum gregorii* MCZ 20143, respectively. (**B**) and (**D**) show the patching result using *I *=* *1 for *S. kirkii* and *S. gregorii*, respectively. *S. kirkii* patches best with *I *=* *0, but *S. gregorii* patches best for *I *=* *1. In these cases, it may be necessary to patch some specimens separately, and recombine the dataset prior to sliding. Specimens displayed in dorsal aspect, with anterior to the right. BMNH, Natural History Museum, London, UK; MCZ, Museum of Comparative Zoology, Cambridge, MA, USA.

Ideally, the same inflate value would be used to patch all specimens. In practice, this may not be possible due to the complexity of the structure and the magnitude of phenotypic disparity (Fig. 23). If this is the case, subsets of specimens can be patched with different inflate values. We argue that more accurate placement of surface points is a far more biologically sound characterization of morphology than spurious placement. The coordinate data from all specimens can then be slid together, to minimize bending energy globally (Fig. 4, cell 5B).

### Partial mesh removal 

During the projection of surface points from the template onto the specimen, surface points tend to be projected onto the first surface they encounter. This situation often occurs with CT scans due to the presence of internal surfaces onto which surface points can be “stuck” inside the external mesh surface. One way to avoid this is to remove internal surfaces using Geomagic Wrap. External elements can be selected, then this selection can be inverted and all the internal surfaces deleted.

**Fig. 24:**
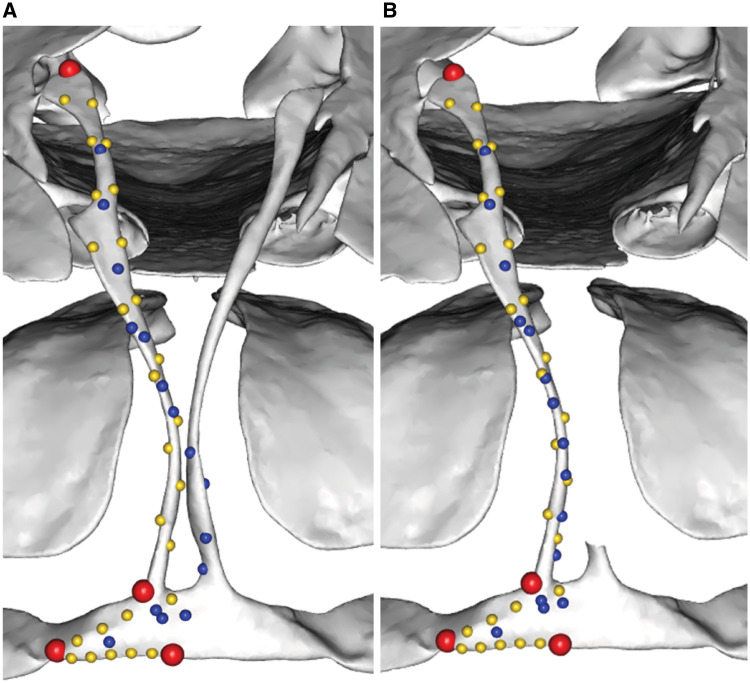
Patching success can be affected by nearby material. Here, the premaxilla (anterior aspect) of the salamander *Bolitoglossa adspersa* ZMB 71710 is being patched, with landmarks (red points) and curves (yellow points) defining the target surface. (**A**) Surface points (blue) can fall outside of the desired region, instead landing on nearby material. (**B**) Removing nearby material from the mesh can result in the surface points patching onto the correct material. Once the patching step has been completed, the original, complete mesh can be used for subsequent steps. ZMB, Zoological Museum of Berlin, Berlin, Germany.

Another potential issue involves external surfaces which are adjacent to the surface targeted for patching. As a solution, problematic areas of the mesh can be removed (Fig. 24). Once surface points are correctly patched on the modified surface scan, patched data can be saved and the mesh can be replaced with the unaltered mesh in order to proceed to the sliding step (Fig. 4, cell 5C).

### Piecemeal patching 

**Fig. 25:**
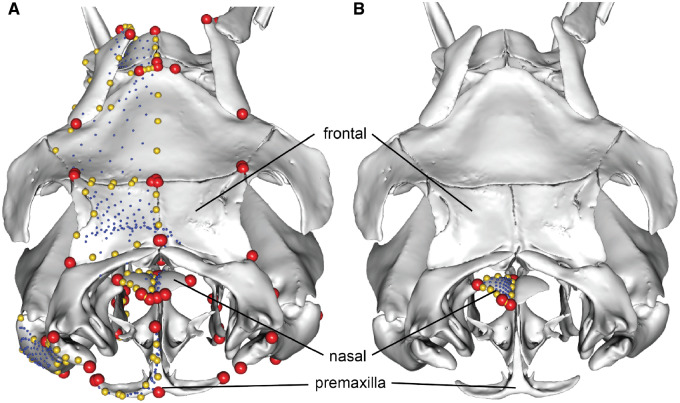
Global and piecemeal patching of surface points on the skull of *Bitis* ZMB 16732. (**A**) Surface points after patching based on the position of all landmarks (red) and curve points (yellow). Note unequal and erroneous placement of surface (blue) points on the nasal and frontal bones. (**B**) Surface points after patching based on the position of landmarks and curve points of the nasal bone. Note the equal distribution of surface points entirely on the nasal bone. (**C**) Final landmark and semilandmark data after merging localized patching across cranial partitions. Note the equal distribution of surface points within regions and lack of erroneous placement of surface points. ZMB, Zoological Museum of Berlin, Berlin, Germany.

For complex anatomical structures, such as skulls, the quality of patching may suffer from attempting to map the surface points on structures based on all landmarks and curves. With the skull of snakes (Watanabe et al., 2019), for example, the placement of surface points on the premaxilla, nasal, and the frontal was uneven and erroneously placed on the other side (Fig. 25A). In contrast, when patching is performed on individual regions or small group of neighboring regions, then the placement of patching improves considerably for those specific regions (Fig. 25B). We recommend a piecemeal patching protocol where individual regions are patched separately and subsequently combined to create a single dataset comprising all patched regions. Because surface points can fall onto the incorrect regions, piecemeal patching makes it easier to visually confirm all surface points were correctly placed. In addition, different parameters for the patching procedure can be used for each region. We found that convex surfaces generally required higher inflate values than concave surfaces (e.g., occipital region, parietal required *I* ∼ 1 and palatal surfaces required *I* ∼ 0 for the clade-wide caecilian study (Bardua et al. 2019), and intraspecific caecilian datasets required *I *=* *0.3 for dorsal [convex] surfaces and *I *=* *0.05 for ventral [concave] surfaces (Marshall et al. 2019)). Increasing or reducing the number of landmarks and curve points used for mapping the template surface points within a localized region (e.g., cranial element) may yield differences in the placement of surface points (Fig. 4, cell 5C).

### Face inversion

Due to fewer landmarks anchoring the mapping of the template surface points onto meshes, a potential issue that arises from piecemeal patching is that the “placePatch” function may have difficulty identifying the orientation of the surface points with respect to the polygon faces of the mesh. Consequently, surface points may be placed on the reverse side of the polygons for some specimens. When this occurs, we suggest patching with an additional region to prevent inversion of faces. For instance, the frontal and parietal may be patched together if the “placePatch” function has difficulty placing surface points on either the frontal or the parietal separately. Alternately, including just the landmarks and curves from additional regions also seems to prevent face inversion. For example, to patch the frontal, the vomer landmarks and curves could be retained on the specimens and template to act as anchors (without the vomer itself being patched). We found that altering the number of landmarks used to map the template surface points onto meshes of specimens within a localized region did not resolve the issue of face inversion (Fig. 4, cell 5D).

## Sliding and alignment

### Overview

Following the patching step, all curve and surface points are slid. This allows these points to be positioned “optimally,” maximizing geometric or biological correspondence across all semilandmarks. The sliding step is important, as the initial arbitrary placement of semilandmarks can impose strong statistical artefacts (Gunz and Mitteroecker 2013). Curves are slid along their tangent vectors, and surface points within their tangent planes, and they are slid either to minimize bending energy or Procrustes distance (Bookstein 1991, 1997; Andresen et al. 2000; Bookstein et al. 2002; Gunz et al. 2005, 2009). Sliding datasets exhibiting large morphological variation using either bending energy or Procrustes distance has shown both alignment criteria to yield results with negligible differences, while these two sliding approaches can create large differences in results for datasets exhibiting small morphological variation (Perez et al. 2006). For these latter datasets, it may be necessary to investigate the impacts that alignment criteria have on results. For sliding 3D data, there are two functions in the *Morpho* R package: “slider3d” (sample-wide relaxing of semilandmarks) and “relaxLM” (relaxing a reference configuration against a target). For detailed descriptions and examples of these sliding procedures, see Schlager (2017).

### Adjusting stepsize for sliding curves

**Fig. 26:**
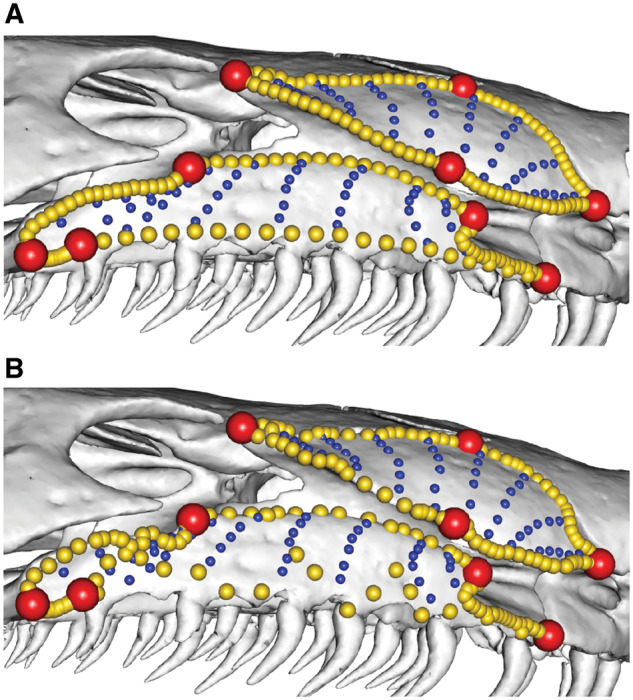
The effect on curves of adjusting the stepsize parameter during the sliding step. During the sliding step, the amount of sliding can be dampened through use of the stepsize argument. Here, the maxillopalatine bone (lateral aspect) of *Geotrypetes seraphini* BMNH field tag MW4543 has been slid using a stepsize of (**A**) 0.1 and (**B**) 2. The higher stepsize has resulted in the curve semilandmarks deviating from their defined curves. BMNH, Natural History Museum, London, UK.

Curves, when slid, should only slide along their predefined paths, and the amount they slide can be dampened by adjusting the stepsize parameter in the “placePatch” function in *Morpho* (Schlager 2017). We found that if the stepsize parameter was set too high, the curve points sometimes deviated from their correct trajectories (Fig. 26). By decreasing the stepsize value to 0.1 (Bardua et al. 2019; Marshall et al. 2019), this problem was alleviated. However, a small stepsize value limits the amount of movement that all curve and surface points can make, so doing so may limit the extent to which bending energy can be minimized (Fig. 4, cell 6A).

### Piecemeal sliding

**Fig. 27:**
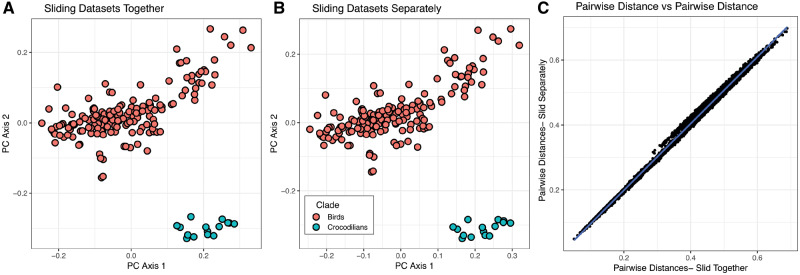
Comparing sliding curve and surface points globally versus sliding subsets separately and combining data. With a dataset of 598 total landmarks, curve and surface points, with 164 bird and 15 crocodilian skulls, we slid curve and surface points to minimize bending energy with two workflows. In one procedure, we slid curve and surface points for all specimens together, then subjected the data to generalized Procrustes analysis (GPA) and PCA (**A**). In the second treatment, we conducted sliding on the two clades separately, then combined them before carrying out GPA and PCA (**B**). These procedures produce nearly identical morphospace distributions. Comparing pairwise distances between taxa for each treatment further demonstrates extremely good fit (**C**).

Under some circumstances, it may be desirable to perform sliding of curve and surface points in a piecemeal fashion as well. For example, when working with fossil specimens with incomplete preservation, it can be useful to deal with one region at a time, patching and sliding curve and surface points for all specimens which preserve that structure. Regions or taxa that have been patched separately then need to be recombined to generate a comprehensive dataset for analyses. Does sliding each region separately influence the global landmark configuration? We compared the effects of performing separate sliding iterations on subsets of the data by placing the same surface patches on two datasets: one composed of 164 bird species and one composed of 15 crocodilian species. We utilized a common template with 292 3D landmarks and curve points and 306 surface points across the skull. We patched crocodilians and birds separately. Next, we slid the curve and surface points to minimize bending energy using two separate procedures: (1) combined the two datasets then applied sliding to all specimens and (2) slid curve and surface points on each dataset separately, then combined them. We calculated the trait covariance matrix for these two treatments and compared correspondence between them using a random skewers analysis with 100,000 iterations. We recovered a correspondence of 0.972 (*P *<* *0.0001), indicating that separate and global sliding of curve and surface points produce nearly identical landmark configurations. This is further illustrated by comparing the results of principal component analyses of these data slid separately to the same data with a global sliding step (Fig. 27). The distribution of taxa in morphospace are nearly identical (Fig. 27A and B). We calculated pairwise Procrustes distances between taxa in both versions of the analysis and plotted the relationship between them (Fig. 27C). A linear regression reveals an extremely strong fit between pairwise distances in the two treatments (*R*^2^ = 0.9975, *P *<* *0.001). Because global versus separate sliding of curve and surface point subsets has little appreciable influence on data distributions, it is expected that sliding regions separately is an appropriate workflow for dealing with high-dimensional 3D surface landmark datasets (Fig. 4, cell 6B).

### Asymmetric sampling of bilaterally symmetric structures

Coordinate data of bilaterally symmetric structures, such as the skull, often comprise landmarks from only one side due to redundant shape information that exists on the other side. In addition, sampling only one side substantially reduces the time required for data collection, which is relevant for the time-intensive acquisition of high-dimensional morphometric data. However, performing generalized Procrustes alignment on one-sided data produces exaggerated shape variation along the anatomical midline while reducing the variation off the midline (Table 5) (Cardini 2016a, 2016b). Following previous studies, we recommend imputation of the missing side through mirroring of the existing side along the midline plane, then removing the mirrored landmarks subsequent to alignment of coordinate data. While previous studies highlighting these artefacts focused on data with only landmarks, here, we investigated which components of high-dimensional coordinates––landmarks, curve points, and surface points—should be mirrored to sufficiently minimize spurious characterization of shape variation.

**Table 5:** Proportional variation of median landmarks relative to total shape variation of right-side landmarks

Dataset	Bird skull	Lizard skull
Right-side only	0.022	0.079
Right-side + left fixed	0.023	0.078
Right-side + left fixed + mirrored curves	0.022	0.072
Right-side + left fixed + mirrored curves and patches	0.020	0.068

To demonstrate the effect of mirroring, we modified an empirical cranial dataset of extant birds (Felice and Goswami 2018) and lizards (Watanabe et al. 2019). Using the mirroring function in the *paleomorph* R package (Lucas and Goswami 2017), we created four datasets: (1) right-side only dataset of landmarks, curve points and surface points; (2) right-side landmarks, curves and surface points with left landmarks (i.e., no left-side curves or surface points) that have all been digitized on actual specimen meshes; (3) right-side landmarks, curves and surface points with left landmarks and mirrored curves; and (4) bilateral pairs of landmarks with mirrored curve and surface points. We then performed generalized Procrustes alignment without sliding the curve and surface points on these datasets, as well as a dataset with coordinate points from one side. To examine the impact of one-sided data, we compared the shape changes associated with PC1 for the four bird datasets as demonstration and compared the proportional variance of landmarks along the midline.

**Fig. 28:**
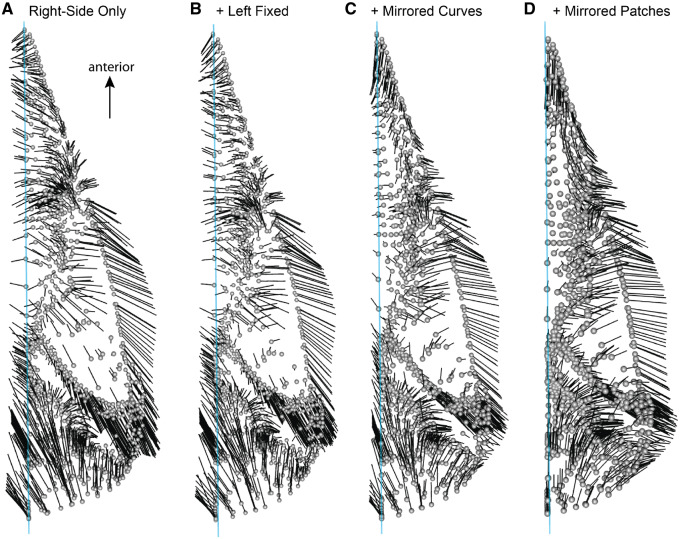
Diagram, in dorsal view, showing shape variation associated with positive PC1 scores of each bird skull dataset highlighting the differences in variation along the midline (blue line). The black lines indicate the magnitude and direction of shape changes from mean shape to shape at maximum PC1 score observed in the sampled specimens for right-side only dataset (**A**); right-side with left landmarks (**B**); right side with left landmarks and mirrored curve points (**C**); and right side with left landmarks and mirrored curve and surface points (**D**). Note the shape variation toward the left side of the skull for landmarks along the median plane (blue line) in datasets aligned without the full set of bilateral landmarks, curve points, and surface points.

As shown in previous studies, the results demonstrate greater shape variation along the midline when data for only one side is aligned under the Procrustean framework, relative to results when both sides are aligned together. Notably, shape changes associated with PC1 in datasets with bilateral pairs of landmarks and curves resemble those of one-side only dataset, exhibiting variation to the left of the skull with more positive PC1 scores (Fig. 28). In contrast, the dataset with entirely two-sided landmarks with mirrored curve and surface points has reduced shape variation along the median line that is oriented in the anteroposterior direction that more accurately reflects biological variation (Fig. 28). Therefore, for high-dimensional data where curve and surface points constitute a substantial portion of the data, they also need to be mirrored (i.e., not only the mirrored or actual landmarks of both sides) to prevent inaccurate or spurious measurement of shape variation. The proportional variance along the median plane relative to total variation of right-side only shape data (without the landmarks and semilandmarks along the median) corroborates this finding, demonstrating elevated proportional variance when only the landmarks and curves are mirrored to create two-sided coordinate data. Given these results, we recommend that landmarks, curves and surface points all have bilateral components through actual digitization or mirroring to prevent artefacts (Fig. 4, cell 6C).

## Conclusions

The collection of semilandmark data (curves and surface points) can be both difficult and time-consuming, so is it worth it? There are many factors to consider when deciding data type, including the intended sample size, complexity of the structure, and the desired resolution of the shape data. Landmarks are considerably faster to collect than semilandmark data, meaning greater taxonomic sampling is easier to achieve. Although pseudolandmarks are also fast to collect, landmarks may be preferred when specific aspects of morphology are of interest, instead of capturing the entire morphology. Thousands of studies to date have successfully captured the shape of structures using 2D or 3D landmark data (e.g., domestic dog crania, Drake and Klingenberg 2010; mouse mandibles, Siahsarvie et al. 2012; caecilian crania, Sherratt et al. 2014; felid vertebrae, Randau et al. 2016; lacertid skulls, Urošević et al. 2018). However, the recent explosion of scan data and the accompanying advances in technology have facilitated the collection of higher-resolution shape information, improving the sampling of shape across a broader range of taxa, and expanding the toolkit for testing a wider range of hypotheses.

Structures with few identifiable landmarks (e.g., limb bones, fused crania) may not be suitable for landmark-only data collection, as this can leave large areas of morphology unsampled. Similarly, sampling over a large, diverse clade may drastically reduce the number of shared landmarks across taxa (Bardua et al. 2019). In these cases, the addition of semilandmarks can greatly improve the characterization of shape. For our caecilian and squamate datasets, landmark-only data can be compared to complete landmark and semilandmark data, by aligning both separately and observing the fit between the two aligned datasets using the “protest” function in the *vegan* R package (Oksanen et al. 2018). A very poor fit was observed between the landmark-only data and the full data for each cranial region for the caecilian and squamate datasets, reflecting the shape information that is lost when only landmarks are used (Tables 3 and 4). Limiting datasets to landmarks would, therefore, mean capturing an exceptionally small amount of the morphological variation across our sample, even within each of the clades of interest. For comparing across clades, the number of Type I and Type II landmarks that can be identified consistently plummets; for example, our estimate of cranial landmarks with unambiguous homology across Tetrapoda numbers approximately 12. Thus, for the purposes of accurately capturing morphological variation, and reconstructing the evolution of form, our results demonstrate that semilandmarks are a vast improvement on landmark-only geometric morphometrics. Accurate analyses of evolutionary processes shaping form require accurate data on morphological variation, and this is not achievable across large clades without moving beyond Type I and Type II landmarks.

Curve semilandmarks expand the quantification of shape to include the morphology of outlines (e.g., bone or fin margins) and ridges. Curve semilandmarks may be sufficient for some structures whose shape is strongly characterized by curves, with relatively conserved surface geometries in between curves (e.g., semi-circular canals, Billet et al. 2015; or bird beaks, Cooney et al. 2017). These data may also be suitable for datasets with moderate levels of surface deformation or incomplete morphologies. The collection of surface semilandmarks can impose strict criteria on the condition of the mesh, since surfaces must be complete and undeformed. Consequently, it may be more practical to collect curve sliding semilandmarks for structures whose surfaces are damaged or incomplete (but whose bone margins or outline information is preserved), but whose morphology would be undersampled using only landmarks.

For studies where the shape of the entire structure is of interest, rather than needing to segregate a structure into component parts, it may be appropriate to capture the shape of surfaces through the use of pseudolandmarks (Vitek et al. 2017). Pseudolandmarks sample over the entire surface of a structure, so the structures must be complete and undeformed. Since they are automatic, these methods facilitate the study of extensive datasets, meaning very large sample sizes can be achieved with relatively little manual input. These methods have been demonstrated using datasets of teeth (Boyer et al. 2011; Vitek et al. 2017) and primate calcanei (Boyer et al. 2015). However, the lack of user control in these methods means that structures cannot be subdivided into different regions, and structures must be treated in their entirety, so questions are limited to looking at gross morphology. Furthermore, complex structures may include areas which should be excluded from shape capture, such as the teeth on a mandible. Since automatic methods cannot distinguish between wanted and unwanted areas of morphology, unwanted regions would have to be manually removed from each structure beforehand. A study comparing the effectiveness of automatic pseudolandmark and semi-automated semilandmark approaches (Gonzalez et al. 2016) found both methods were successful for simple, smooth shapes with high levels of variation across the dataset. However, semiautomatic methods were more successful at discriminating group differences for more complex and irregular shapes (and datasets exhibiting lower levels of morphological variation). This may be because pseudolandmark approaches sample evenly over a structure so cannot focus on specific regions of interest which may be the key to between-group differences (Gonzalez et al. 2016). However, choice of alignment settings can affect the success of pseudolandmark approaches, and increased numbers of pseudolandmarks can improve the detection of group differences, suggesting this approach may be suitable for datasets with small amounts of variation (although sensitivity analyses should be run for each dataset) (Vitek et al. 2017). Pseudolandmark approaches are therefore most appropriate for relatively simple structures when large sample size is desired and biological questions are centered around gross morphology.

**Fig. 29:**
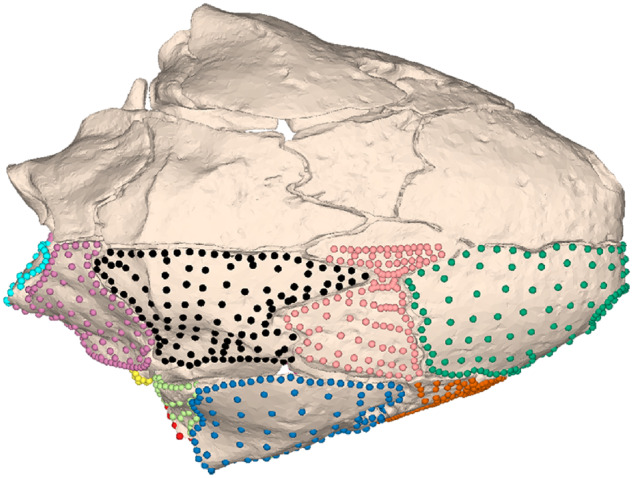
Annotated 3D version of this figure available at: https://sketchfab.com/3d-models/b3db492d35964e0282a81179972e5083. Landmarks and semilandmarks, color coded by the 16 cranial regions defined in Bardua et al. (2019), shown on the caecilian *Siphonops annulatus* BMNH 1956.1.15.88. Regions are as follows: nasal, premaxilla (or nasopremaxilla), and septomaxilla when present, dorsal surface (green); frontal and mesethmoid when present (light pink); parietal (black); squamosal and postfrontal when present (dark blue); maxillopalatine (lateral surface) and prefrontal when present (orange); quadrate (lateral surface) (light green); quadrate (jaw joint articulation) (red); occipital (otic) region of os basale (excluding occipital condyle) (light purple); occipital condyle (aqua); ventral surface of os basale (purple); palatal surface of nasopremaxilla or the anterior projection of the vomer (gold); vomer (white); interdental plate of maxillopalatine (gray); palatine shelf (maxillary plate) of maxillopalatine (hot pink); pterygoid, and/or pterygoid process of quadrate (light blue); stapes (yellow). BMNH, Natural History Museum, London, UK.

**Fig. 30:**
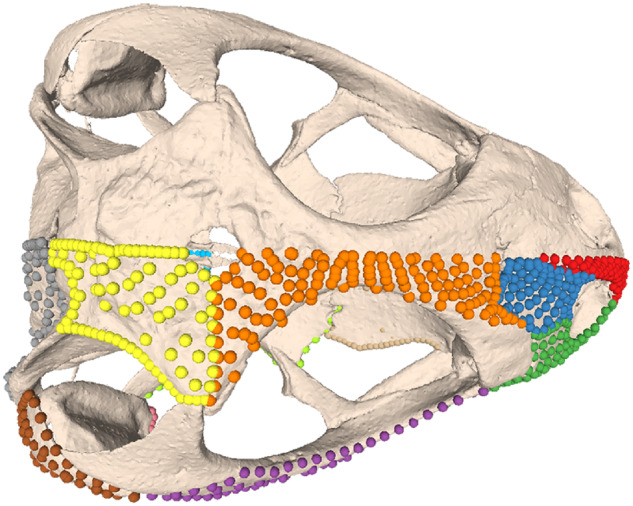
Annotated 3D version of this figure available at: https://sketchfab.com/3d-models/b046136256904b4293b630272f2134b8. Landmarks and semilandmarks, color coded by the 13 cranial regions defined in Watanabe et al. (2019), shown on the lizard *Sceloporus variabilis* FMNH 122866. Regions are as follows: premaxilla (red), nasal (dark blue), maxilla (dark green), jugal (purple), frontal (orange), parietal (yellow), squamosal (brown), jaw joint (pink), supraoccipital (gray), basioccipital (light blue), pterygoid (light green), palatine (tan), and occipital condyle (black). FMNH, Field Museum of Natural History, Chicago, IL, USA.

Surface semilandmark approaches as described here provide an intermediate between lower-density landmark-based studies and extremely high-density pseudolandmark studies. Semilandmarks are able to discriminate group morphology for diverse datasets (Gonzalez et al. 2016). Furthermore, use of semilandmarks allows detection of subtle morphological variation, making them crucial for morphologically restricted studies (e.g., intraspecific or within-population studies). Critically, the method described allows for the demarcation of regions that correspond to homologous structures, for example, the frontal bone or the rostrum (Figs. 29 and 30), meaning that relationships among regions or differential patterns of variation across regions can be assessed. Although the specific sutures defining a region, or even the elements comprising a structure may vary across large (or even small) clades, they can be compared in a biologically meaningful manner using semilandmarks. Therefore, while automated procedures may be suitable for capturing the overall morphology of some structures, semi-automated procedures may be better suited to investigating localized shape variation (Gonzalez et al. 2016). Furthermore, the relationships among regions, that is, integration and modularity, can be examined using this approach, because the surface point positions are informed by landmarks and curves that have been placed with user input, rather than an entirely automated process. Surface semilandmarks also facilitate the warping of one structure’s morphology to another, for use in fossil reconstructions and hypothetical model construction (e.g., Gunz et al. 2009; O’Higgins et al. 2011). Both semilandmark and pseudolandmark approaches, therefore, offer promising and complementary paths forward for comparing across disparate organisms or for comparing structures that may not have many clear landmarks.

Of course, using high-density approaches such as those described here may create issues with data dimensionality, and this effect should be considered and checked in downstream analyses. There is also the additional problem that many existing analytical tools cannot cope with large datasets at present (Adams and Collyer 2017), although new methods are in continuous development to solve these issues (Clavel et al. 2019). One approach that we have implemented is to subsample down to 10–20% of the full landmark and semilandmark dataset and rerun analyses to check consistency of results. For our analyses of trait correlation structure (integration and modularity), we subsampled our datasets of birds, squamates, and caecilians down to 10% of the full dataset and compared results from 100 iterations to that for the full dataset. Results were consistently nearly identical across subsamples and the full datasets (Watanabe et al., 2019; Felice and Goswami 2018; Bardua et al. 2019; Marshall et al. 2019), indicating that they are robust to landmark sampling, but this should be checked separately for every dataset. This effect has also been demonstrated for high-dimensional shape data for musteloid limbs, finding subsampled data lead to the same results (Fabre et al. 2014). Randomly subsampling from the complete dataset (and running analyses iteratively) offers the additional benefit of enabling sampling from the whole of morphology, achieving dimensionality similar to that of landmark-only datasets but without restricting the shape data to sutures and other Type I and Type II landmarks, which tend to be limited to the boundaries of structures.

Biological variation is inherently high-dimensional (Collyer et al. 2015). In order to best reconstruct and examine morphological variation and morphological evolution, it is imperative to accurately measure organismal form. The past few decades have brought extraordinary new abilities to image organisms with more speed and resolution than previously possible, and many current initiatives are focused on digitizing biological diversity at scales that would have been unimaginable when geometric morphometric approaches were first being applied to macroevolutionary questions. These new datasets bring challenges, but they also provide unprecedented opportunities to identify the fundamental rules shaping evolution across disparate species (Goswami et al. 2019). To do so will require further expansion and development of tools that can capture and leverage this new information to its fullest potential. We hope that this practical guide to applying surface sliding semilandmark methods across a wide diversity of forms will prove useful in advancing the fields of quantitative evolutionary and comparative biology toward that goal.
